# Micronutrient intake and status in young vegans, lacto-ovo-vegetarians, pescatarians, flexitarians, and omnivores

**DOI:** 10.1007/s00394-024-03453-4

**Published:** 2024-07-18

**Authors:** Synne Groufh-Jacobsen, Christel Larsson, Claire Margerison, Isabelle Mulkerrins, Dagfinn Aune, Anine Christine Medin

**Affiliations:** 1 Department of Nutrition and Public Health, Faculty of Health and Sport Science, University of Agder, Universitetsveien 25, Kristiansand, 4630 Norway; 2 Department of Food and Nutrition, and Sport Science, Faculty of Education, University of Gothenburg, PO Box 300, Gothenburg, SE-405 30 Sweden; 3 Institute for Physical Activity and Nutrition, Deakin University, Geelong, 3216 Australia; 4 Department of Epidemiology and Biostatistics, School of Public Health, Imperial College London, London, UK; 5 Department of Nutrition, Oslo New University College, Oslo, Norway; 6 Department of Research, The Cancer Registry of Norway, Oslo, Norway

**Keywords:** Diet, Dietary assessment, Nutrient intake, Nutrition status, Plant-based diet, Youth

## Abstract

**Purpose:**

Whether youth who follow plant-based diets in Nordic countries meet their dietary needs for micronutrients remains unclear. This study aims to evaluate micronutrient intake and status in Norwegian youth following vegan, lacto-ovo-vegetarian, pescatarian, flexitarian and omnivore diets.

**Methods:**

Cross-sectional design, with healthy 16-to-24-year-olds (*n* = 165). Participants were asked to complete a questionnaire and four 24-hour dietary recalls. Dried blood spots (DBS) and spot-urine samples were collected for analysis of methyl malonic acid (MMA) (*n* = 65), haemoglobin (Hb) (*n* = 164) and urinary iodine concentration (UIC) (*n* = 163).

**Results:**

Vegans reported highest habitual supplement usage of multivitamin (58%), B_12_ (90%) and macroalgae consumption (32%), while flexitarians reported highest habitual usage of omega-3 supplements (56%). For daily supplement usage, vegans reported highest use of multivitamins (42%), B_12_ (79%), iodine (37%) and iron (63%). Increased risk of inadequate intake (energy-adjusted) were found for vitamin D (60% within lacto-ovo-vegetarians), selenium (70% within lacto-ovo-vegetarians, 65% within omnivores), and iodine (63% within vegans). Median MMA levels suggest low risk of insufficient B_12_ status across all groups (MMA 0.04‒0.37µmol/l) and 2% had MMA levels indicating possible B_12_ deficiency and 8% had elevated levels. Median Hb levels indicated low risk of anemia across all groups (≥ 12.0 females, ≥ 13.0 g/dl males), though 7% had Hb values indicating risk of mild anemia and 4% risk of moderate anemia. The median UIC indicates mild iodine deficiency in all groups (UIC < 100 µg/l), except vegans, who were moderately iodine deficient (UIC < 50 µg/l).

**Conclusions:**

Our study indicated that the participating youth had low risk of inadequate intake of most micronutrients, partly due to high supplement usage. However, for iodine, vitamin D, and selenium higher risk of inadequate intake was found. UIC corroborated the low iodine intake among vegans. Thus, we suggest iodine status of youth in Norway should be monitored, especially among young fertile women who omits dietary iodine sources, until a mandatory iodine fortification program is implemented. Furthermore, we suggest that food education on how to secure sufficient nutrients from food in general should be provided to the Norwegian youth population, especially how to secure adequate intake of vitamin D, selenium and iodine.

**Supplementary Information:**

The online version contains supplementary material available at 10.1007/s00394-024-03453-4.

## Introduction

Current evidence recommends limiting the consumption of processed and red meat for a reduced risk of noncommunicable diseases [1–6], and several reviews and studies have linked a diet with reduced meat consumption and vegetarian diets with a variety of health benefits [7–10]. By securing a well-planned plant-based diet containing a variety of fruits, vegetables, whole grains, legumes, nuts and seeds, the diet can be nutritionally adequate and promote health during all stages of the life course, although evidence of long-term effects is limited in critical stages of the life course, such as the youth period [11–13]. However, in addition to having a well-planned diet, supplementation of key nutrients is needed if following a vegan diet, if not obtaining these micronutrients through fortified foods. Particularly B_12_ is of concern for those who follow vegan diets, which is found mainly in foods such as meat, poultry, seafood, eggs, and dairy products [14], an essential vitamin that needs to be secured through the diet. Furthermore, the key nutrients that are of concern in plant-based diets are vitamin B_12_, vitamin D, calcium, zinc, selenium, iodine, vitamin A, riboflavin, and iron [15]. Although the nutrients of concern depend on the types of plant-based diets followed and the fortification strategy in that country. Thus, when omitting animal-source foods from the diet, it is necessary to substitute with food sources that are equivalent in most nutrients to avoid an increased risk of nutrient deficiencies over time. Ensuring adequate intake of the above mentioned key nutrients is also of utmost importance in the youth period due to increased nutritional requirements due to growth [16, 17].

Previously in Nordic countries, risk of inadequate intake of vitamin D, folate, calcium, and selenium have been reported in national dietary data among adults [18]. Furthermore, risk of inadequate iodine intake has also been documented in the general population across European countries [19], especially among fertile women, and is also widespread among the general population in Norway [20]. Thus, risk of inadequate intake of certain micronutrients are also present in the general population in Nordic countries. Currently, there is insufficient knowledge on micronutrient intake and status of youth who follows different plant-based diets in Nordic countries [11]. Thus, this study aims to evaluate the micronutrient intake and status of 16-to-24-year-olds in Norway following different plant-based diets compared to omnivores.

## Methods

### Study design and recruitment

Between September 2021 and March 2022, 16-to-24-year-olds were recruited through convenience sampling and snowball sampling in the Agder area in southern Norway. Participants were recruited to participate in a cross-sectional project called VeggiSkills-Norway. The study eligibility criteria were: (1) able to read and understand Norwegian; (2) 16–24 years of age; (3) no acute or chronic illness; (4) currently not pregnant/lactating; (5) not having children; (6) having followed a vegan, lacto-ovo-vegetarian, pescatarian, flexitarian, or omnivore diet for a minimum of 6 months prior to participation, and no current plan to alter their dietary practice; (7) possibility of physical attendance at the University of Agder, Kristiansand, Norway.

After inclusion, participants completed an electronic questionnaire comprising 183 items (Supplemental Table 5) that evaluated the sociodemographic characteristics of the participants, health and lifestyle habits, sleeping habits and sedentary behavior and food literacy competencies. A dietary screener assessing consumption frequency of 33 selected food groups (“MyFoodMonth 1.1”) [21] was included in the questionnaire as well as questions concerning food habits in the previous six months (meal frequencies, supplements, dietary practice).

After filling out the electronic questionnaire onsite, participants were asked to complete the first out of four non-consecutive 24-hour dietary recalls (interview-administered). Subsequently, participants provided a non-fasted dried blood sample and a urinary sample for analysis of nutritional status biomarkers, and body composition measurements were also performed. After the study visit, participants completed the remaining three self-administered non-consecutive 24-hour dietary recalls. Participants were asked to report their intake of all foods, beverages, and supplements consumed the previous day in a Norwegian version of the web-based image-assistant dietary assessment tool, Measure Your Food on One Day (Myfood24) [22].

### Sample size calculation

A priori, the sample size was determined based on a previously conducted study in young Swedish vegans and omnivores that detected group differences with 30 participants in each diet group [23]. Our final sample size was determined by the recruitment feasibility since the study took place during the COVID-19 pandemic.

### Classification into dietary groups

Participants were classified into vegan, lacto-ovo-vegetarian, pescatarian, flexitarian and omnivore diet groups based on animal-sourced food groups included in their diet the previous six months, as previously described [24]. Out of the 165 participants, 19 were classified as vegans, with no inclusion of animal-source foods [1, 25], 20 participants were classified as lacto-ovo-vegetarians, including dairy / eggs to varying degree [1, 25] and 30 participants were classified as pescatarians, as they additionally reported eating seafood (regardless of the reported intake of dairy / eggs) [1]. Twenty-five participants were classified as flexitarians as they self-reported having a flexitarian diet in the electronic questionnaire and also reported consuming less than two servings per week of meat or poultry (any type) in the dietary screener [26]. Lastly, a total of 71 participants were classified as omnivores if they reported two or more servings of meat per week in the dietary screener.

### 24-hour dietary recalls

Participants were instructed to complete four 24-hour dietary recalls on random non-consecutive days, over a period of eight weeks using Myfood24 [22, 27]. Participants were asked to report their intake of all foods, beverages, and supplements consumed in the previous 24-hours based on the multiple pass method. The first 24-hour dietary recall was completed at the study site and carried out as an interview. To ensure 24-hour dietary recalls on weekends and weekdays, participants received an email with a link to complete recalls on different days. A maximum of two reminders were used to complete the dietary recalls, with one-week intervals. Participants who completed a minimum of one 24-hour dietary recall were included in the present study. In total, all the 165 participants completed day 1, 79% day 2, 60% day 3, and 44% day 4 of the 24-hour dietary recalls.

Myfood24 uses the Norwegian Food Composition Database [28] to calculate the energy and nutrient intake. If participants had consumed foods or beverages not listed in the database, they were instructed to choose an alternative but similar item (e.g., if consumed a plant-based substitute for yoghurt not present in the database, they were encouraged to choose another similar plant-based substitute available). It was also possible to report food items and amount consumed in an open text field at the end. All items reported in the open text option were processed manually. For recipes/foods not listed in the database (e.g., vegan mayonnaise), manual calculation was performed. In Norway, only plant-based substitutes to dairy products are allowed to be fortified, at present. If the participants reported consumption of plant-based substitutes to dairy products in the open text field, the researcher manually entered a plant-based substitute to dairy products available in the database with similar nutrient content. Furthermore, for foods reported with no similar food product available in the database, the product label was used to obtain a similar energy and macronutrient profile of the product. For example, if a participant reported consuming a vegan product mainly consisting of vegetable oil, water and sugar, the raw ingredients were entered to obtain the similar energy and macronutrient profile of the portion consumed.

### Supplement use

Dietary supplements by 24-hour use was assessed using Myfood24 by collecting information on brand/type and amount/dose consumed the previous day in an open text question. Amount/dose of supplements reported in the 24-hour recalls are used when describing the total intake (including foods and supplements) in Tables 1, 2, 3, 5 and 4. The use of supplements reported in the 24-hour recalls is also reported as n (%) in Table 1.

Dietary supplements by habitual use the previous six months were assessed in the dietary screener (“MyFoodMonth 1.1”) [21], using a dichotomous variable, yes/no for the use of selected supplements including multivitamin, vitamin B_12_, iodine, omega-3 fatty acids, vitamin D, iron, folate, nutritional yeast and macroalgae. A string alternative ‘other supplements’ was also available. Habitual supplement use is presented in Table 1 as n (%).

### Physical activity level

The physical activity level (PAL) of the participants was classified based on the reported frequency of moderate to vigorous-intensity activities in the previous six months using age specific PAL values [29, 30]. See Supplemental Table 5 for the items used to assess self-reported PAL. A low PAL value of 1.55 was assigned to participants who selected either of the options ‘never’ or ‘less than once a month’ or ‘once a week’ for the previous six-month period. Furthermore, a moderate PAL value of 1.7 was assigned if the option ‘two – three times a week’ was selected. A high and very high PAL value of 1.85-2.0 was assigned if options 4–5 times a week or ‘almost every day’ were selected.

### Anthropometric measurements

Height measurement was carried out for all participants, barefoot, and standing to the nearest 0.1 cm using a portable stadiometer according to a measurement protocol (Sect. 7.2.2 in the protocol, page.72) [31]. Body weight was measured barefoot and in light clothing, in a non-fasted state, using a *Tanita* MC780 bioelectric impedance analyzer (Tokyo, Japan) and using the Tanita user manual. Body mass index (BMI) was calculated using height and weight (body weight (kg) /m^2^).

### Blood sampling and analysis of methyl malonic acid (MMA) and haemoglobin (hb)

Whole blood was collected in non-fasted state by dried blood sampling (DBS), a biosampling method where capillary blood was obtained by a fingerprick using a disposable lancet. Complete DBS cards were allowed to dry overnight (maximum 12 h) in a room without light exposure, before being stored in a sealed aluminium bag with a desiccant pack at -20 degrees Celsius (°C) until analysis. Methylmalonic acid (MMA) was analyzed from the whole blood (VITAS™ Analytical Services, Norway). A sufficient volume of blood for analysis of MMA was available for 65 participants. MMA was extracted from 8 mm DBS punches using an isotope dilution GC-MS assay. Four punches were eluted in an aqueous alkaline solution containing stable isotope-labelled MMA prior to derivatization using propyl chloroformate. The liquid extraction of derivatized MMA was performed before injection into the GC-MS system. The GC-MS system utilized was an Agilent 6890 GC (Agilent Technologies, Palo Alto, CA, USA) equipped with an Agilent 5973 N mass-selective detector operated in EI-SIM mode. Separation was performed on a Zebron ZB-AAA GC column supplied by Phenomenex Inc (Torrance, CA, USA) and quantification was performed against a 5-point standard curve prepared from standards with known concentrations.

Haemoglobin (Hb) was measured from the same finger prick as the whole blood for DBS, using Hemocue® HB801 Analyzer and Microcuvettes (Sweden). In total 164 participants provided blood for analysis of Hb.

### Collection of urinary samples and analysis of urinary iodine concentration (UIC)

Participants provided a non-fasted spot urine sample collected at random times throughout the day, using a 100 ml Vacuette® urine breaker (Greiner Bio-One, Kremsmünster, Austria). Thereafter, a subsample of urine was withdrawn from the urine breaker into a 9.5 ml Vacuette® urine tube (Greiner Bio-One, Kremsmünster, Austria). The urine samples were stored at -20 degrees Celsius (℃) until analysis at the Hormone Laboratory, Oslo University Hospital, Norway. In total, 163 participants provided enough urine volume to perform UIC analysis.

After thawing of the urine samples, 250 µl of each urine sample was diluted into 1 ml of ammonium persulfate for oxidation of interference substances. The UIC was determined using the Sandel-Kolthoff reaction method (colorimetric) based on the catalytic effect of iodine on the red/ox reaction between arsenic and cerium, after digestion of the samples. Samples were analyzed using microtiter plates (Delfia Microtitration Plate from Wallace Oy, Denmark) using 405 nm for Victor^3^ (Perkin Elmer, Finland). Method blank samples (H_2_O) were used, using reference material, KIO_3,_ Sigma-Aldrich prepared in the same manner as the respective samples. The lower limit of detection was 0.01 µmol/l, and the lower limit of quantification was 0.2 µmol/l.

### Evaluation of biomarkers

To indicate risk of insufficient B_12_ status, the functional marker MMA (µmol/l) was assessed. The reference interval for adequate MMA levels at 0.04‒0.37 µmol/l was used, for elevated MMA levels the cut-off at > 0.27 µmol/l was used and for risk of B_12_ deficiency the cut-off at > 0.37 µmol/l was used [32, 33].

To assess risk of anemia, WHO diagnostic criteria using Hb concentration was used [34]. Low risk of anemia was defined as Hb levels ≥ 12.0 g/dl for the women and ≥ 13.0 g/dl for men. Risk of mild anemia as Hb levels in the range 11.0–11.9 g/dl for women and 11.0–12.9 g/dl for men. Risk of moderate anemia as Hb levels in the range 8.0–10.9 g/dl and risk of severe anemia as Hb levels lower than 8.0 g/dl for both women and men.

To evaluate iodine status, WHO epidemiological criteria was used [35]. The iodine status was evaluated based on the median UIC, and the median UIC < 20 µg/l was considered as risk of severe iodine deficiency, median UIC at 20–49 µg/l as risk of moderate iodine deficiency, median UIC at 50–99 µg/l as risk of mild iodine deficiency, median UIC at 100–199 µg/l as adequate iodine status, median UIC at 200–299 µg/l as above the iodine requirements and median UIC ≥ 300 µg/l as risk of excessive iodine intake.

### Statistical analysis

For data analysis the software used was IBM SPSS statistics version 29 (IBM Corp., Armonk, NY, United States) and Excel® (Microsoft 365). The calculation of the micronutrient intake of each supplement was performed manually in Excel®.

The data distribution was evaluated using tests for normality, and in addition visual inspection of the histogram and Q-Q plots were checked. Residual plots were also examined. To test for differences between groups, the ANOVA test (parametric), the Mann-Whitney U test (nonparametric, two groups), and the Kruskal-Wallis one-way ANOVA test (nonparametric, more than two groups) with correction for multiple comparisons were used. For categorical variables, cross-tabulation using the Pearson Chi-square or Fisher exact test was used to test for differences between groups. The adjusted p-value < 0.05 (two-sided) was used for the significance level for all tests.

### Energy adjustment of micronutrient intake

Energy adjustment was carried out according to the principle of nutrient density [36] and individual energy requirements:

$$\begin{array}{*{20}{c}} {Average{\text{ }}micronutrient} \\ {intake{\text{ }}measured{\text{ }}in} \\ {units{\text{ }}per{\text{ }}MJ} \end{array}{\text{ }}=\frac{\begin{gathered} total~average~ \hfill \\ micronutrient\,intake \hfill \\ ~measured~in~units~per~day~ \hfill \\ \end{gathered} }{\begin{gathered} reported~total~energy \hfill \\ ~intake~in~MJ~per~day \hfill \\ \end{gathered} } \times 1000$$

Subsequently, the micronutrient intake was adjusted for energy according to individual estimated basal metabolic rate (BMR) and individual estimated PAL value [37]. The BMR in MJ per day was estimated using Henry’s [38]. For the calculation of the total energy requirement, the estimated BMR was multiplied by individual PAL values for a low, moderate, and high activity level (see method section, physical activity).

$$\begin{array}{*{20}{c}}{\text{Estimated micronutrient intake}}\\{\text{per day according to}}\\{\text{individual energy requirement in MJ}} \end{array} = \begin{gathered}\left( \begin{gathered}{\text{Estimated micronutrient}} \hfill \\{\text{intake in mg per MJ}} \times \hfill \\{\text{estimated total energy}} \hfill \\{\text{requirement in MJ}} \hfill \\ \end{gathered} \right) \hfill \\ \end{gathered}$$

We compared energy-adjusted intakes for each individual with the average requirements (AR) for females and males in the age group 16–17 years and 18–24 years with values in the Nordic Nutrition Recommendations 2023 (NNR2023) [39].

### Multivariable adjustments

Quantile regression was performed to adjust the 25th, 50th and 75th percentiles of the micronutrient intake for potential influencing confounding factors [40]. The dietary groups were included as fixed factors using dummy variables. Further, age and gender were identified as potentially influencing confounding factors as these variables varied significantly between groups. Therefore, age and gender were included in the quantile regression model as covariates. Adjusted 95% confidence intervals (95%CI) for the medians were obtained from the quantile regression.

A previous publication found significant differences between diet groups in the general nutrition knowledge (GNKQ) score [24]. Since this variable also varied significantly between groups it was of relevance to try to adjust for it. However, due to the presence of groups with fewer than 30 participants within the plant-based diet groups it was not possible to adjust for the GNKQ score variable in a multivariable adjustment. Hence, we opted to adjust for age and gender. As we could not adjust for GNKQ as a covariate in the multivariable-adjustment model, a sensitivity analysis was carried out evaluating whether energy-adjusted total micronutrient intake (including food and supplements) varied according to poor, moderate and high GNKQ level (Table 4). Participants with a total sum score with less than 60% correct answers were considered poor, participants with 60–79% correct answers were considered moderate, and participants with 80–100% correct answers were considered high. Categorization into the different levels of GNKQ and calculation of the GNKQ score has previously been described elsewhere [24].

## Results

### Characteristics of study participants

The characteristics of the participants are presented in Table 1. The mean ± SD age of the participants was 21 ± 2 years. Omnivores had the lowest percentage of women versus the highest percentage among lacto-ovo-vegetarians (*P* = 0.005).

In total, 72% of all participants reported habitually consuming any type of supplement in the previous six months, in which pescatarians reported the lowest percentage (60%) and vegans the highest (95%) (*P* = 0.003). For habitual supplement usage within the dietary groups, vegans reported the highest habitual supplement use of multivitamins (58%) and of B_12_ (90%) and lowest usage was reported among omnivores (23% multivitamin and 9% B_12_, respectively) (*P* = 0.030 and *P* < 0.001). Furthermore, the highest habitual consumption of macroalgae (32%) was reported among vegans, compared to zero consumption in the other diet groups. Habitual usage of omega-3 supplements also varied between the groups, with the highest consumption among flexitarians (56%) and lowest among vegans (21%).

For supplement usage reported in the 24-hour dietary recalls, 58% of all participants reported consuming any type of dietary supplement. Reported 24-hour supplement use of multivitamin, iodine, B_12_ and iron varied between groups, in which vegans reported the highest usage of all the respective supplements (multivitamin 42%, iodine 37%, B_12_ 79%, iron 63%) and lowest usage among omnivores (< 15% for each supplement).

**Table 1:** Participant characteristics of 16–to-24-year-olds in Norway following different dietary practices

	All^†^ *n* = 165		Vegans^†^ *n* = 19		Lacto-ovo-vegetarians^†^ *n* = 20		Pescatarians^†^ *n* = 30		Flexitarians^†^ *n* = 25		Omnivores^†^ *n* = 71		*P*
	*n*	%	*n*	%	*n*	%	*n*	%	*n*	%	*n*	%	
Sex													**0.005**
Female^‡^	125	76	13	68	19	95	26	87	22	88	45	63	
Male^‡^	40	24	6	32	1	5	4	13	3	12	26	37	
Parental educational level													0.56
Parental educational level ≤ 16 years^‡^	72	48	7	44	10	63	12	43	13	57	30	44	
Parental educational level > 16 years^‡^	79	52	9	56	6	38	16	57	10	44	38	56	
Habitual, all supplements^‡^	119	72	18	95	18	90	18	60	21	84	44	62	**0.003**
Habitual, multivitamin supplements^‡^	56	34	11	58	9	45	12	40	8	32	16	23	**0.030**
Habitual, vitamin D supplements^‡^	37	22	6	32	6	30	5	17	6	24	14	20	0.62
Habitual, folate supplements^‡^	4	2	–	–	1	5	1	3	–	–	2	3	0.88
Habitual, macroalgae consumption^‡^	6	4	6	32	–	–	–	–	–	–	–	–	**< 0.001**
Habitual, iodine supplements^‡^	5	3	1	5	–	–	3	10	–	–	1	1	0.11
Habitual, omega-3 supplements^‡^	65	39	4	21	5	25	9	30	14	56	33	47	**0.045**
Habitual, B_12_ supplements^‡§^	47	29	17	90	9	45	7	23	8	32	6	9	**< 0.001**
Habitual, B_12_, injections ^‡^	2	1	1	5	–	–	–	–	1	4	0	0	0.14
Habitual, iron, supplements^‡^	28	17	5	26	5	25	6	20	5	20	28	17	0.22
24 h, all supplements^‡‖^	95	58	16	84	12	60	14	47	16	64	37	52	0.07
24 h, multivitamin supplements^‡^	20	12	8	42	1	5	6	20	3	12	20	12	**< 0.001**
24 h, iodine supplements^‡‖‖‖^	32	19	7	37	4	20	7	23	7	28	7	10	**0.039**
24 h, B_12_ supplements^‡^	48	29	15	79	4	20	9	30	10	40	10	14	**< 0.001**
24 h, iron supplements^‡^	43	26	12	63	5	25	7	23	9	36	10	14	**< 0.001**
	Mean	SD^‖‖^	Mean	SD^‖‖^	Mean	SD^‖‖^	Mean	SD^‖‖^	Mean	SD^‖‖^	Mean	SD^‖‖^	
Age, years^‡¶^	21	2	22	2	21	2	22^*^	2	22	2	20^‖^	2	**0.010**
Body mass index, kg/m^2‡^	23	4	22	4	23	5	24	3	22	4	24	3	0.32
Total general nutrition knowledge score (GNKQ)^‡,a^	48	9	49	11	49	9	50	8	52^*^	6	46^‖^	9	**0.025**
	Median	IQR^‖‖^	Median	IQR^‖‖^	Median	IQR^‖‖^	Median	IQR^‖‖^	Median	IQR^‖‖^	Median	IQR^‖‖^	
Absolute energy intake, MJ^‡^	7	3	7	3	6^*^	2	8^‖^	2	6	2	7^‖^	3	**0.002**

^†^Percentage presented within each diet group; ^‡^Test for the difference between categorical variables using cross tabulation with Fisher-Exact Test (two-sided), for continuous parametric variables one-way ANOVA with Bonferroni Post Hoc test with correction for multiple comparisons was used and Kruskal Wallis one-way ANOVA test for non-parametric continuous variables, values with different superscript indicate statistical significant differences (^‖,*^); ^§^Habitual B_12_ supplement use includes nutritional yeast (B_12_ rich) (12 vegans, 2 lacto-ovo vegetarians, 2 pescatarians, 1 flexitarian); ^‖‖‖^ Macroalgae not reported by 24-hour usage; ^‖‖^ SD = Standard deviation, IQR = Inter quartile range; ^¶^ In the post-hoc test pescatarians differed from omnivores (*P* = 0.043); ^a^ GNKQ score has previously been described in detailed elsewhere ^(24)^, included as a background variable, maximum possible points 67; Statistically significant values between the dietary groups < 0.05 are given in bold

### Reported energy intake

Differences in reported energy intake was found between lacto-ovo-vegetarians (6 MJ/day) compared to omnivores (7 MJ/day, *P* = 0.011), and between lacto-ovo-vegetarians compared to pescatarians (8 MJ/day, *P* = 0.008) (Table 1).

### Micronutrient intake

The energy-adjusted micronutrient intake solely from foods and the total intake from both foods and supplements are presented in Tables 2 and 3. For absolute micronutrient intake (not energy-adjusted) solely from foods and total absolute intake (not energy-adjusted) from both foods and supplements are presented in Supplemental Tables 1 and 2.

**Table 2:** Adjusted intake of vitamins by average intake from 24-hour dietary recalls in 16-to-24-year-olds following different dietary practices

Energy-adjusted^¶^	All *n* = 165			Vegans *n* = 19			Lacto-ovo-vegetarians *n* = 20			Pescatarians *n* = 30			Flexitarians *n* = 25			Omnivores *n* = 71			*P*
	50th	25th, 75th	95%CI	50th	25th, 75th	95%CI	50th	25th, 75th	95%CI	50th	25th, 75th	95%CI	50th	25th, 75th	95%CI	50th	25th, 75th	95%CI	
Vit A, RE/d, food^‡^	578	413, 816	511, 661	421^*^	250, 595	256, 595	586	461, 729	496, 664	670^‖‖^	440, 968	463, 820	521	380, 805	439, 727	644^‖‖^	418, 911	511, 661	**0.032**
Vit A, RE/d, total^‡^	915	623, 1439	807, 1025	850	411, 1514	411, 1514	795	629, 1042	701, 1017	1096	660, 1530	766, 1373	1066	652, 1417	724, 1285	890	636, 1377	791, 1042	0.65
Niacin, NE/d, food^‡^	19	15, 27	18, 21	18	15, 25	15, 25	15^*^	10, 24	10, 23	19	14, 23	15, 21	19	14, 25	15, 25	21^‖‖^	17, 33	19, 24	**0.023**
Niacin, NE/d, total^‡^	23	16, 40	20, 26	34^*^	17, 60	17, 60	16^‖‖^	11, 32	12, 27	21	15, 32	16, 24	25	17, 41	19, 35	23	18, 41	20, 28	**0.028**
Vit B_2_, mg/d, food^‡^	2	2, 3	2, 2	2	1, 2	1, 2	2	1, 4	2, 3	2	1, 2	2, 2	2	1, 2	2, 2	2	2, 3	2, 2	0.15
Vit B_2_, mg/d, total^‡^	2	2, 4	2, 3	3	2, 5	2, 5	2	2, 4	2, 4	2	1, 3	2, 3	2	2, 3	2, 3	2	2, 4	2, 3	0.51
Vit B_12_, µg/d, food^‡^	5	3, 8	5, 6	1^*^	1, 1	1, 1	4^‖‖^	2, 8	2, 7	5^‖‖^	3, 8	4, 7	6^‖‖^	3, 8	4, 7	7^‖‖^	5, 10	5, 8	**< 0.001**
Vit B_12_, µg/d, total^‡^	7	4, 10	6, 8	11	3, 18	3, 18	5	2, 11	2, 9	6	4, 9	5, 8	7	6, 10	6, 9	7	5, 10	5, 9	0.52
Folate, µg/d, food^‡^	347	271, 468	331, 370	531^*^	381, 628	381, 628	389	328, 472	335, 452	350^‖‖^	275, 504	292, 486	339^‖‖^	269, 417	284, 401	318^‖‖^	252, 379	272, 350	**< 0.001**
Folate, µg/d, total^‡^	390	294,534	358, 425	565^*^	381, 805	381, 805	428	338, 574	346, 536	440	289, 531	320, 514	519^*^	337, 766	343, 559	337^‖‖^	268, 414	358, 425	**<0.001**
Vit D, µg/d, food^‡^	4	2, 7	3, 4	2^*^	1, 3	1, 3	3	1, 5	1, 5	5^‖‖^	2, 10	3, 9	4^‖‖^	2, 10	2, 8	4^‖‖^	2, 7	3, 6	**0.004**
Vit D, µg/d, total^‡^	9	3, 25	7, 12	22	3, 39	3, 39	6	1, 40	2, 35	12	5, 18	6, 16	9	4, 27	5, 23	9	3, 22	5, 13	0.68
Vit E, α-TE, food^‡^	16	13, 21	15, 17	18	15, 22	15, 22	14	12, 19	12, 19	18	14, 24	16, 22	15	12, 19	13, 19	15	12, 20	13, 17	0.05
Vit E, α-TE, total^‡^	19	14, 29	18, 21	22	16, 39	16, 39	17	13, 23	13, 21	23	16, 31	17, 31	19	15, 32	15, 29	18	13, 25	17, 21	0.23
Vit C, mg/d, food^‡^	109	67, 158	95, 118	154	94, 226	94, 226	110	90, 145	93, 136	93	56, 141	72, 111	116	76, 156	85, 138	104	40, 163	73, 123	0.06
Vit C, mg/d, total^‡^	118	77, 216	108,142	226	111, 312	111, 312	124	90, 184	92, 181	108	74, 246	88, 152	138	96, 196	108, 188	109	52, 203	89, 148	0.07
*Multivariable adj.* ^¶^	50th	25th, 75th	95%CI	50th	25th, 75th	95%CI	50th	25th, 75th	95%CI	50th	25th, 75th	95%CI	50th	25th, 75th	95%CI	50th	25th, 75th	95%CI	
Vit A, RE/d, food	574	402, 794	356, 878	384	225, 590	93, 706	612	437, 634	345, 902	651	430, 891	349, 971	541	408, 752	301, 862	598	526, 866	553, 874	
Vit A, RE/d, total	924	642, 1378	476, 1325	813	396, 1674	299, 1259	751	695, 980	281, 1202	1039	646, 1385	508, 1498	1055	663, 1266	627, 1482	925	657, 1336	472, 1362	
Vit B_3_, NE/d, food	20	15, 27	13, 32	19	15, 23	10, 31	17	11, 29	9, 28	19	15, 25	10, 31	20	15, 26	12, 32	22	17, 33	14, 33	
Vit B_3_, NE/d, total	24	16, 39	6, 46	39	20, 49	23, 42	17	14, 30	2, 32	21	16, 31	4, 41	25	18, 37	12, 44	23	17, 38	9, 41	
Vit B_2_, mg/d, food	2	2, 3	1, 3	2	1, 2	1, 3	2	1, 3	1, 3	2	1, 2	1, 3	2	1, 2	1, 3	2	2, 3	1, 3	
Vit B_2_, mg/d, total	2	2, 4	1, 4	3	2, 5	2, 5	2	2, 4	1, 5	2	2, 3	0, 4	2	2, 3	1, 4	2	2, 4	1, 4	
Vit B_12_, µg/d, food	6	2, 8	3, 9	1	1, 2	0^a^, 4	4	2, 7	1, 6	6	3, 8	2, 9	6	4, 8	3, 9	7	5, 10	4, 10	
Vit B_12_, µg/d, total	7	4, 10	4, 11	12	3, 19	7, 16	6	3, 11	2, 10	6	4, 9	2, 11	7	6, 9	4, 12	7	5, 10	3, 11	
Folate, µg/d, food	344	268, 451	218, 471	531	399, 593	410, 655	370	327, 481	258, 467	355	269, 493	230, 485	346	273, 409	247, 469	324	263, 373	218, 430	
Folate, µg/d, total	396	304, 514	208, 551	579	397, 781	425, 723	398	346, 571	256, 520	433	285, 520	272, 586	507	339, 620	383, 650	343	269, 424	207, 464	
Vit D, µg/d, food	4	2, 7	1, 6	2	1, 3	0^a^, 5	3	1, 5	0^a^, 6	5	2, 9	1, 8	3	1, 9	0^a^, 6	5	2, 7	1, 8	
Vit D, µg/d, total	9	4, 24	0^a^, 20	25	3, 38	12, 36	5	2, 33	0^a^, 16	10	5, 18	0^a^, 19	7	3, 25	0^a^, 17	9	4, 20	0^a^, 19	
Vit E, α-TE, food	16	13, 21	11, 21	18	16, 21	12, 23	14	13, 20	9, 19	19	14, 23	12, 24	15	12, 19	10, 20	16	12, 20	11, 20	
Vit E, α-TE, total	19	14, 29	12, 27	22	15, 37	13, 30	18	13, 21	9, 27	23	16, 31	13, 31	18	15, 29	9, 26	19	13, 25	11, 27	
Vit C, mg/d, food	109	66, 156	49, 163	147	97, 217	80, 206	112	83, 138	51, 172	87	57, 125	18, 147	111	80, 124	54, 167	105	51, 168	46, 163	
Vit C, mg/d, total	115	73, 225	36, 182	242	110, 348	163, 317	124	87, 172	40, 190	103	68, 231	23, 174	132	92, 223	52, 194	110	61, 215	43, 169	

Abbreviations: 50th, 25th, 75th = percentiles, 95%CI = 95% confidence interval of the 50th percentile, foods = solely from foods, total = including foods and supplements; ^‡^Test for the difference using Kruskal Wallis test using one-way ANOVA with correction for multiple comparisons, values with different superscript indicate statistically significant differences from post hoc test (^‖‖^,^*^); Statistically significant values between the dietary groups < 0.05 are given in bold; ^¶^Energy-adjusted according to the nutrient density approach and individual energy requirement using Henry equation for estimation of basal metabolic rate and with individual physical activity level, the multivariable adjustment variable is in addition adjusted for gender and age; ^a^ 95%CI with zero needs to be interpreted with caution due to high degree of uncertainties

**Table 3:** Adjusted intake of mineral and trace elements by average intakes from 24-hour dietary recalls in 16-to-24-year-olds following different dietary practices

Energy-adjusted^¶^	All *n* = 165			Vegans *n* = 19			Lacto-ovo- vegetarians *n* = 20			Pescatarians *n* = 30			Flexitarians *n* = 25			Omnivores *n* = 71			*P*
	50th	25th, 75th	95%CI	50th	25th, 75th	95%CI	50th	25th, 75th	95%CI	50th	25th, 75th	95%CI	50th	25th, 75th	95%CI	50th	25th, 75th	95%CI	
Magnesium, mg/d, food^‡^	437	370, 525	416, 460	647^*^	503, 777	503, 777	441^‖‖^	358, 541	367, 513	434^‖‖^	379, 522	387, 473	430^‖‖^	366, 499	373, 496	426^‖‖^	365, 488	392, 450	**0.002**
Magnesium, mg/d, total^‡^	466	388, 588	445, 505	706^*^	528, 826	528, 826	485	394, 569	369, 554	453^‖‖^	382, 524	393, 512	496	414, 584	430, 554	445^‖‖^	381, 557	415, 500	**0.006**
Calcium, mg/d, food^‡^	985	767, 1304	932, 1067	941	728, 1279	728, 1279	989	809, 1111	815, 1111	959	752, 1502	769, 1375	905	649, 1229	728, 1279	1040	800, 1343	933, 1173	0.61
Calcium, mg/d, total^‡^	1030	798, 1431	945, 1111	974	765, 1332	765, 1332	989	828, 1111	866, 1111	959	767, 1502	800, 1375	940	691, 1364	792, 1279	1054	870, 1462	956, 1288	0.68
Iron, mg/d, food^‡^	13	11, 17	13, 14	19^*^	13, 21	13, 21	13^‖‖^	10, 17	11, 16	13^‖‖^	10, 17	11, 15	13^‖‖^	12, 15	12, 14	13^‖‖^	10, 15	12, 14	**0.002**
Iron, mg/d, total^‡^	15	12, 20	14, 17	27^*^	17, 36	17, 36	15	11, 22	11, 19	15	12, 19	13, 18	16	13, 26	13, 22	14^‖‖^	11, 17	13, 15	**0.005**
Zinc, mg/d, food^‡^	12	10, 15	11, 13	12	9, 14	9, 14	11^*^	9, 12	9, 12	11	10, 15	10, 14	11	10, 13	10, 13	13^‖‖^	11, 16	12, 14	**0.012**
Zinc, mg/d, total^‡^	13	11, 17	12, 15	18^*^	11, 32	11, 32	12^‖‖^	9, 14	9, 14	14	10, 18	10, 15	13	10, 17	11, 16	14	11, 17	12, 15	**0.047**
Selenium, µg/d, food^‡^	52	38, 67	47, 55	42	29, 55	29, 55	37^*^	28, 46	29, 41	54^‖‖^	41, 75	42, 63	55^‖‖^	35, 76	44, 73	55^‖‖^	45, 68	50, 61	**0.002**
Selenium, µg/d, total^‡^	57	41, 87	52, 63	66	30, 133	30, 133	37	29, 74	30, 61	62	42, 98	47, 86	73	52, 117	52, 94	56	45, 69	52, 63	0.06
Iodine, µg/d, food^‡^	109	76, 166	100, 122	50^*^	41, 58	41, 58	95^‖‖^	76, 136	76, 134	123^‖‖^	93, 171	106, 152	109^‖‖^	80, 232	90, 157	122^‖‖^	82, 190	107, 147	**< 0.001**
Iodine, µg/d, total^‡^	125	80, 216	114, 148	56	41, 294	41, 294	110	77, 155	80, 145	154	106, 305	109, 235	157	94, 254	104, 225	124	82, 200	109, 165	0.08
*Multivariable adjusted* ^¶^	50th	25th, 75th	95%CI	50th	25th, 75th	95%CI	50th	25th, 75th	95%CI	50th	25th, 75th	95%CI	50th	25th, 75th	95%CI	50th	25th, 75th	95%CI	
Magnesium, mg/d, food	439	373, 520	317, 574	619	471, 752	491, 755	481	369, 521	362, 582	433	389, 501	298, 578	439	375, 494	335, 575	429	365, 501	312, 549	
Magnesium, mg/d, total	477	390, 585	320, 640	665	535, 806	516, 814	487	381, 567	348, 594	443	392, 519	284, 604	481	426, 554	361, 630	460	378, 544	322, 591	
Calcium, mg/d, food	989	764, 1472	753, 1361	937	744, 1202	629, 1318	980	835, 1126	685, 1385	998	773, 1414	705, 1387	936	674, 1187	675, 1322	1036	826, 1345	770, 1380	
Calcium, mg/d, total	1013	800, 1366	752, 1370	981	768, 1328	611, 1421	979	841, 1119	644, 1428	997	790, 1464	639, 1435	982	750, 1297	671, 1421	1086	892, 1479	802, 1459	
Iron, mg/d, food	13	11, 16	10, 17	19	14, 21	15, 23	14	11, 16	11, 18	13	10, 18	10, 17	14	12, 15	11, 17	13	10, 15	10, 16	
Iron, mg/d, total	15	12, 21	10, 21	27	17, 35	22, 32	16	11, 23	13, 21	15	12, 18	9, 20	16	12, 22	12, 21	14	11, 18	10, 19	
Zinc, mg/d, food	12	10, 14	9, 17	12	10, 14	8, 17	12	10, 13	9, 13	12	10, 15	8, 18	12	11, 13	9, 17	13	11, 15	10, 18	
Zinc, mg/d, total	13	11, 18	9, 19	19	11, 33	15, 24	12	10, 15	12, 18	14	10, 18	10, 20	13	11, 17	9, 18	14	12, 15	10, 18	
Selenium, µg/d, food	52	38, 66	32, 74	35	30, 52	15, 55	38	27, 45	19, 54	55	40, 74	34, 76	54	37, 72	38, 62	54	45,69	37, 71	
Selenium, µg/d, total	58	41, 85	30, 87	64	29, 134	37, 90	40	29, 79	15, 62	61	44, 88	33, 88	71	52, 111	48, 97	55	47, 74	32, 77	
Iodine, µg/d, food	113	75, 163	69, 157	47	36, 60	0^a^, 95	95	79, 138	46, 140	125	95, 160	70, 175	109	83, 166	66, 158	125	87, 183	80, 167	
Iodine, µg/d, total	131	84, 231	58, 203	50	37, 281	0^a^, 125	118	79, 143	41, 190	151	104, 293	65, 231	161	94, 239	93, 237	131	86, 204	60, 198	

Abbreviations: 50th, 25th, 75th = percentiles, 95%CI = 95% confidence interval of the 50th percentile, foods = solely from foods, total = including foods and supplements;^‡^Test for the difference using Kruskal Wallis one-way ANOVA with correction for multiple comparisons, values with different superscript indicate statistically significant differences from post hoc test (^‖‖^,^*^); Statistically significant values between the dietary groups < 0.05 are given in bold; ^¶^Energy-adjusted according to the nutrient density approach and individual energy requirement using Henry equation for estimation of basal metabolic rate and with individual physical activity level, the multivariable adjustment variable is in addition adjusted for gender and age; ^a^95%CI with zero needs to be interpreted with caution

### Multivariable adjustment of micronutrient intake

Multivariable adjustment (age and gender) of the energy-adjusted micronutrient intake is presented in Tables 2 and 3. Multivariable adjustment (age and gender) did not significantly affect the median intake of the energy-adjusted micronutrients solely from foods or total intake (including foods and supplements). Furthermore, after performing the multivariable adjustment, the 95%CI were found to be overlapping for nearly all the medians across the diet groups, except for total intake of iron (including foods and supplements), in which vegans still differed from the other diet groups.

Energy-adjusted total micronutrient intake (including foods and supplements) according to poor, moderate and high GNKQ level is presented in Table 4. Total energy-adjusted intakes (including foods and supplements) of vitamin A (*P* = 0.005), folate (*P* = 0.013), vitamin D (*P* = 0.006), vitamin C (*P* = 0.021) and iron (0.003) differed between the GNKQ levels, in which higher median intakes were found among those with high GNKQ level compared to those of poor GNKQ level.

**Table 4:** Energy-adjusted total intake of micronutrients by poor, moderate, and high general nutrition knowledge (GNKQ) level (*n* = 165)

Energy-adjusted intakes^¶^	Poor GNKQ^§^ *n* = 22			Moderate GNKQ^§^ *n* = 92			High GNKQ^§^ *n* = 51			*P*
	50th	25th, 75th	95%CI	50th	25th, 75th	95%CI	50th	25th, 75th	95%CI	
Vit A, RE/d, total^‡^	752^*^	548, 997	553, 958	886	606, 1731	791, 1047	1008^‖‖^	766, 1510	853, 1285	**0.005**
Vit B3, NE/d, total^‡^	21	13, 36	13, 34	23	16, 44	19, 29	23	16, 35	19, 30	0.69
Vit B2, mg/d, total^‡^	2	1, 3	1, 3	2	2, 4	2, 3	2	2, 3	2, 3	0.62
Vit B12, µg/d, total^‡^	6	4, 12	4, 12	7	4, 10	6, 8	7	5, 18	6, 9	0.82
Folate, µg/d, total^‡^	327^*^	278, 415	280, 414	383	268, 554	332, 452	478^‖‖^	354, 545	385, 504	**0.013**
Vit D, µg/d, total^‡^	4^*^	1, 9	1, 9	9	4, 25	7, 12	15^‖‖^	4, 29	8, 22	**0.006**
Vit E, α-TE, total^‡^	17	13, 32	13, 31	19	14, 26	17, 21	21	15, 32	18, 28	0.43
Vit C, mg/d, total^‡^	89^*^	64, 143	74, 142	114	66, 229	97, 149	148^‖‖^	106,230	112, 190	**0.021**
Magnesium, mg/d, total^‡^	421	377, 531	380, 522	451	374, 599	430, 488	514	433, 590	473, 554	0.07
Calcium, mg/d, total^‡^	985	752, 1749	765, 1721	1049	793, 1370	939, 1139	1020	826, 1540	890, 1231	0.87
Iron, mg/d, total^‡^	13^*^	9, 17	9, 16	15	11, 21	14, 16	17^‖‖^	13, 29	14, 19	**0.003**
Zinc, mg/d, total^‡^	12	9, 17	10, 17	13	11, 18	12, 14	15	11, 16	12, 15	0.53
Selenium, µg/d, total^‡^	55	31, 68	32, 67	55	39, 91	47, 63	57	45, 94	52, 76	0.33
Iodine, µg/d, total^‡^	118	76, 213	76, 206	123	83, 377	109, 145	148	77, 264	107, 191	0.44

Abbreviations:50th, 25th, 75th = percentiles, 95%CI = 95% confidence interval of the 50th percentile, GNKQ = General Nutrition Knowledge ^§^ Poor GNKQ = total GNKQ sum score < 60% correct, moderate GNKQ = total GNKQ sum score correct answers between 60–79%, high GNKQ = sum score 80–100% correct answers (previously described in details elsewhere ^(24)^); ^‡^Test for the difference using Kruskal Wallis one-way ANOVA with correction for multiple comparisons, values with different superscript indicate statistically significant differences from post hoc test (^‖‖^,^*^); Statistically significant values between the dietary groups < 0.05 are given in bold; ^¶^ Energy-adjusted according to the nutrient density approach and individual energy requirement using Henry equation for estimation of basal metabolic rate and with individual physical activity level

### Proportion of individuals below average requirements (AR)

The proportion of individuals with total energy-adjusted micronutrient intake (including foods and supplements) below AR is presented in Table 5. The proportions of individuals below AR using absolute micronutrient intake solely from foods and total intake (including foods and supplements) (not energy-adjusted) is presented in Supplemental Table 3.

**Table 5:** Proportion of individuals below average requirement (AR) among 16-to-24-year-olds following different dietary practices

Including food and supplements					All^†^		Vegans^†^		Lacto-ovo- vegetarians ^†^		Pescatarians^†^		Flexitarians^†^		Omnivores^†^		
	Female 16–17 years	Males 16–17 years	Females 18–24 years	Male 18–24 years	n	%	n	%	n	%	n	%	n	%	n	%	*P*
Energy-adjusted vitamin A, RE/d^‡,¶^	500	600	540	630	28	17	6	32	2	10	3	10	3	12	14	20	0.29
Energy-adjusted niacin, NE/MJ^‡,¶^	1.3	1.3	1.3	1.3	18	11	1	6	6	30	4	13	1	4	6	9	0.07
Energy-adjusted riboflavin, mg/d^‡,¶^	1.3	1.3	1.3	1.3	19	12	2	11	3	15	3	10	2	8	9	13	0.96
Energy-adjusted vitamin B_12,_ µg/d^‡,¶^	3.1	3.2	3.2	3.2	21	13	4	21	7	35	5	17	1	1	4	6	**0.004**
Energy-adjusted folate, µg/d^‡,¶^	240	250	250	250	18	11	1	5	0	0	3	10	3	12	11	16	0.37
Energy-adjusted vitamin D, µg/d^‡,¶^	7.5	7.5	7.5	7.5	75	46	8	42	12	**60**	11	37	11	44	33	47	0.60
Energy-adjusted vitamin E, α-TE/d^‡,¶^	9	10	8	9	1	1	0	0	0	0	0	0	0	0	1	1	1.00
Energy-adjusted vitamin C, mg/d^‡,¶^	75	85	75	90	41	25	1	5	4	20	8	27	3	12	25	35	**0.031**
Energy-adjusted magnesium, mg/d^‡,¶^	200	240	240	280	5	3	1	5	1	5	1	3	0	0	2	3	0.75
Energy-adjusted calcium, mg/d^‡,¶^	980	980	870	870	55	33	7	37	7	35	12	40	9	36	20	28	0.78
Energy-adjusted iron, mg/d^‡,¶^	9	9	9	7	8	5	1	5	2	10	2	7	1	4	2	3	0.57
Energy-adjusted zinc, mg/d^‡,¶^	10.2	11.7	8.1	10.6	17	10	2	11	4	20	3	10	2	8	6	9	0.65
Energy-adjusted selenium, µg/d^‡,¶^	55	70	60	70	94	**57**	9	47	14	**70**	14	47	11	44	46	**65**	0.14
Energy-adjusted iodine, µg/d^‡,¶^	100	110	120	120	70	42	12	**63**	9	45	12	40	9	36	28	40	0.40

^†^Percentage presented within each diet group below average requirement, in which each individuals are compared against The Nordic Nutrition Recommendation 2023 according to the average requirement value specific for their age and gender; ^‡^Test for the difference (categorical variables) using cross tabulation with Fisher-Exact test (two-sided); ^¶^Energy-adjusted according to the nutrient density approach and individual energy requirement using Henry equation for estimation of basal metabolic rate and with individual physical activity level; Statistically significant values between the dietary groups < 0.05 are given in bold and values with proportion equal to or larger than 50% are given in bold

Risk of inadequate micronutrient intake (including foods and supplements) were found for vitamin D (60% within lacto-ovo-vegetarians < AR), selenium (70% within lacto-ovo-vegetarians and 65% within omnivores < AR), and iodine (63% within vegans < AR) after energy-adjustment. Between the dietary groups, a significant difference in the percentage below AR was found for total intake of vitamin B_12_ (35% within lacto-ovo-vegetarians vs. 4% within flexitarians, *P* = 0.004) and for total intake of vitamin C (35% within omnivores vs. 5% within vegans, *P* = 0.031) after energy-adjustment.

The proportion of individuals with total energy-adjusted micronutrient intake (including foods and supplements) below AR according to GNKQ level is presented in Supplemental Table 4. Between the different GNKQ levels, energy-adjusted total micronutrient intakes (including foods and supplements) of vitamin A (*P* < 0.001), vitamin D (*P* = 0.012), vitamin C (*P* = 0.021) and zinc (*P* = 0.006) varied significantly, with highest proportion below AR among those with poor GNKQ level compared those with high GNKQ level. Risk of inadequate micronutrient intake based on GNKQ level was found for vitamin D in those with poor GNKQ level (73% < AR), and for selenium, for all GNKQ levels (64% within poor level, 57% within moderate level, 55% within high level).

### Biomarkers of nutritional status

#### Methylmalonic acid (MMA) as a functional marker of B_12_ status

For the total group (*n* = 65), the median (25th, 75th ) level of MMA was 0.19 µmol/l (1.16, 0.23), and all the dietary groups had median MMA levels indicating lower risk of B12 deficiency, with no difference between the groups (Table 6). Indication of elevated MMA levels (> 0.27 µmol/l) were found in 8% of the participants, with no difference between the groups. Increased risk of B_12_ deficiency (MMA > 0.37 µmol/l) was found in one participant who adhered to a pescatarian diet.

**Table 6:** Nutritional status indicator of B_12_, anemia (haemoglobin) and iodine status in 16-to-24-year-olds following different dietary practices (*n* = 165)

Biomarker	All^†^		Vegans^†^		Lacto-ovo-vegetarians^†^		Pescatarians^†^		Flexitarians^†^		Omnivores^†^		*P*
	50th	25th, 75th	50th	25th, 75th	50th	25th, 75th	50th	25th, 75th	50th	25th, 75th	50th	25th, 75th	
MMA, µmol/l, group total^‖,§^	0.19	1.16, 0.23	0.19	0.19, 0.23	0.20	0.18, 0.24	0.19	0.15, 0.22	0.18	0.16, 0.23	0.22	0.13, 0.23	0.87
Hb, g/dl, group total^‖,§^	14	13, 15	15	13, 15	13^*^	12, 14	14	13, 15	14	13, 15	15^‡^	13, 15	**0.012**
Hb, g/dl, females	14	13, 15	14	13, 15	13	12, 14	14	13, 14	14	13, 15	14	13, 15	< **0.001**
Hb, g/dl, males	15	15, 16	15	14, 16	–	–	16	14, 17	16	15	15	15, 16	
UIC, µg/l, group total^‖,§^	75	44, 114	20^*^	16, 53	82^‡^	41, 110	83^‡^	40, 133	71^‡^	39, 152	77^‡^	56, 116	**< 0.001**
	*n*	%	*n*	%	*n*	%	*n*	%	*n*	%	*n*	%	
*B12 status indicator*													
Elevated MMA, > 0.27 µmol/l^¶^	5	8	1	10	0	0	2	14	1	7	1	7	0.82
Risk of B_12_ deficiency, > 0.37 µmol/l^¶^	1	2	0	0	0	0	1	7	0	0	0	0	
*Iron status indicator (anemia)*													0.72^¶^
Mild anemia, < 12 g/dl^¶^	11	7	1	5	2	10	2	7	2	8	4	6	
Moderate anemia, < 11 g/dl^¶^	6	4	2	10	1	5	0	0	0	0	3	4	
Severe anemia, < 8 g/dl^¶^	0	0	0	0	0	0	0	0	0	0	0	0	
*Iodine status indicator*													
UIC, < 50 µg/l^¶^	50	31	12	71	6	30	9	30	8	32	15	21	**0.005**
UIC, < 100 µg/l^¶^	114	70	16	94	14	70	18	60	17	68	49	69	0.15

^†^Percentage presented within each diet group (for descriptive purpose); ^‖^MMA = methyl malonic acids (all *n* = 65, vegans *n* = 10, lacto-ovo-vegetarians *n* = 12, pescatarians *n* = 14, omnivores *n* = 15), Hb = haemoglobin (all *n* = 164, vegans *n* = 19, lacto-ovo-vegetarians *n* = 20, pescatarians *n* = 30, flexitarians *n* = 24, omnivores *n* = 71), UIC = urinary iodine concentration (all *n* = 163, vegans *n* = 17, lacto-ovo-vegetarians *n* = 20, pescatarians *n* = 30, flexitarians *n* = 25, omnivores *n* = 71); ^§^ Test for the difference using Kruskal Wallis one-way ANOVA with correction for multiple comparisons, values with different superscript indicate statistically significant differences from post hoc test (^*,‡^); ^¶^Test for the difference (categorical variables) using cross tabulation with Fisher-Exact Test (two-sided); Statistically significant values between the dietary groups < 0.05 are given in bold

### Haemoglobin (hb) as a marker of anemia

The median Hb (*n* = 164) in the different dietary groups indicates low risk of anemia (Table 6). At the group level, males had higher median Hb levels (25th, 75th ) than females, 15 g/dl (15,16) vs. 14 g/dl (13,15) (*P* < 0.001), both indicating low risk of anemia. Lacto-ovo-vegetarians had the lowest median Hb (25th, 75th ) at 13 g/dl (12, 14) and omnivores the highest Hb level at 15 g/dl (13,15) (*P* = 0.007). None of the participants had Hb values that indicated risk of severe anemia (< 8 g/dl), 4% had Hb values that indicated risk of moderate anemia (< 11 g/dl) and 7% had Hb values that indicated risk of possible mild anemia (< 12 g/dl).

### Urinary iodine concentration (UIC) as a marker of iodine status

For the total group (*n* = 163), the median UIC (25th, 75th ) was 75 µg/l (44, 114), indicating that 70% had risk of mild iodine deficiency (median UIC < 100 µg/l) and 31% had risk of moderate iodine deficiency (median UIC < 50 µg/l) Table 6. Vegans had the lowest median UIC at 20 µg/l (16, 53), compared to all other diet groups, flexitarians at 71 µg/l (39, 152) (*P* < 0.001), omnivores at 77 µg/l (56, 116) (*P* < 0.001), lacto-ovo-vegetarians at 82 µg/l (41, 110) (*P* = 0.018) and pescatarians at 83 µg/l (40, 133) (*P* = 0.001).

## Discussion

In this study, micronutrient intake and status were evaluated in healthy 16-to-24-year-olds following vegan, lacto-ovo-vegetarian, pescatarian, flexitarian, and omnivore diets (*n* = 165). Most participants reported using a dietary supplement habitually (72%) the previous six months. The micronutrient intakes that persisted below AR when including foods and supplements (energy-adjusted) were vitamin D (lacto-ovo-vegetarians), selenium (lacto-ovo-vegetarians and omnivores), and iodine (vegans), indicating risk of inadequate intake of some micronutrients among youth with plant-based or omnivorous diets. The findings from the dietary intake data (including food and supplements), showed that intakes of B_12_ and iron were above AR for all groups, indicating low risk of inadequate intakes. This was supported by objective blood markers of MMA and Hb, which also indicated low risk of B_12_ deficiency and low risk of anemia across all dietary groups. The UIC indicated that risk of mild iodine deficiency was prevalent in all dietary groups indicated by a median UIC < 100 µg/l, except vegans who were found to be at risk of being moderately deficient in iodine, indicated by a median UIC < 50 µg/l.

### Micronutrient adequacy of plant-based and omnivorous diets among youth

In our study, vegans did not meet AR solely through foods (energy-adjusted) for vitamin A, vitamin B_12_, vitamin D, selenium, and iodine, indicating an increased risk of inadequate intake of these nutrients without supplementation. Furthermore, none of the other diet groups had median energy-adjusted intakes solely through foods that met AR for vitamin D and selenium. Moreover, solely through foods, the lacto-ovo-vegetarians and flexitarians did not obtain AR for iodine.

From total intake including food and supplements (energy-adjusted), both youth with plant-based and omnivorous diets faced nutritional challenges in meeting AR, particularly selenium, vitamin D and iodine. The proportions of participants with total intake including food and supplements (energy-adjusted) below AR for vitamin D was 46%, with highest proportion among the lacto-ovo-vegetarians (60% < AR). Furthermore, 57% of the individuals had intakes below AR for selenium (70% within lacto-ovo-vegetarians and 65% within omnivores < AR). For iodine, 42% of the individuals had intakes below AR (63% within vegans < AR). Therefore, the risk of inadequate intake of these nutrients were present in these subgroups in our study. However, it needs to be notated that the AR values for selenium was revised and increased in the NNR2023, which might impact the percentage categorized with inadequate intake [39]. These findings highlight the importance of providing sufficient food education to youth on how to meet their dietary needs through foods. Also, the importance of consuming fortified foods or supplementing for certain micronutrients when following plant-based diets to compensate for the lower presence of some micronutrients in the diet. Our findings are consistent with previous research [11], including previous findings in young Swedish vegans [23]. Furthermore, our findings are also partly in coherence with a newly published study among German children and adolescents, in which the study found differences in biomarkers between the dietary groups who followed vegan, vegetarian and omnivore diets, but the study indicated no specific nutritional risk between the diet groups [41].

Since none of the dietary groups in this present study was able to obtain AR for vitamin D without supplementation, and only 22% of the young participants reported habitual consumption of vitamin D supplements, there is a risk of insufficient vitamin D status over time. Especially during the winter season without exposure to the sun in Norway, which necessitates the importance of adequate dietary intake of vitamin D. Our findings of inadequate vitamin D intake are consistent with national surveys among Norwegian young adults aged 18 to 29 years conducted a decade ago [42].

### B_12_ intake and marker of status

In our study, the participating vegans did not obtain an adequate B_12_ intake solely through foods (energy-adjusted) which has also been shown in previous research [43]. Although in Norway most plant-based dairy substitutes are fortified with B_12_. However, total median B_12_ intake (energy-adjusted) including foods and supplements was well above AR in all dietary groups. The MMA level measured in DBS in our study also corroborated the lower risk of inadequate B_12_ intake, as 2% had possibly B12 deficiency with no significant difference between groups. Furthermore, 8% were found to have elevated MMA levels, but no significant differences between groups. Although MMA is the most sensitive single marker of B_12_, it is recommended to evaluate several markers of B_12_ for evaluating status [44]. Our finding of B_12_ intake above AR in the young vegans in our study, is explained by high usage of B_12_ supplements (90% habitually and 79% by 24-hour usage), which supports previous finding of the importance of supplementing with B_12_ in addition to using fortified foods when following plant-based diets, especially vegan diets [43, 45]. A concern is still expressed for young people following plant-based diets, especially young vegans who are not sufficiently educated on supplement usage or how to obtain nutrients from plant-based foods and fortified foods. Youth who are not sufficiently educated can be particularly vulnerable to inadequate intake of certain essential micronutrients as B_12_.

Our findings of adequate B_12_ intake are in coherence with previous studies, among young Germans who adhered to different plant-based diets and among Norwegian adults who adhered to vegan, vegetarian, and pescatarian diets [41, 45]. However, a previous review (including 40 studies with infants, children, adolescents, pregnant women, adults and elderly from several countries) has shown that vegans who did not supplement with vitamin B_12_ were at higher risk of B_12_ deficiency compared to vegetarians, based on serum B_12_ [43]. Due to our sample size, we were not able to perform analysis comparing B_12_ supplement users and non-supplement users within the dietary groups.

### Iron intake and marker of status

In the present study, the energy-adjusted iron intake solely from foods and total iron intake (including foods and supplements) indicated low risk of inadequate intake. However, the findings did not account for the bioavailability of iron from plant sources, and a recently published study demonstrated that the uptake of iron and zinc from some plant-based meat substitutes could be poorer than anticipated [46]. On the other hand, higher intakes of vitamin C and organic acids in fruits and vegetables are known to improve iron absorption and could mitigate lower iron absorption from plant foods, if consumed together [47]. In our study, 7% had Hb values indicating mild anemia and 4% moderate anemia, although none of the dietary groups was found to differ in risk of anemia, which is consistent with previous studies investigating iron status in those following plant-based diets and omnivores [11, 41, 48]. On the contrary, a previous literature review found a higher prevalence of iron deficiency among adult vegans compared to vegetarians (from 10 different countries), in which 8 of 13 included studies used Hb as a marker of iron status. However, it should be noted that the included studies used different iron deficiency cutoff criteria [48].

### Iodine intake and marker of status

The findings of median iodine intake including foods and supplements (energy-adjusted) below AR among the young vegans and lacto-ovo-vegetarians corresponds with the concentration of iodine measured in the urine, with UIC < 50 µg/l in the vegans and < 100 µg/l in the lacto-ovo-vegetarians. Our findings of UIC indicating mild to-moderate iodine deficiency are in line with previous findings in adult vegans and lacto-ovo-vegetarians in Norway [49, 50]. Furthermore, our findings are also in agreement with a newly published review reporting that vegans and vegetarians who did not consume iodine-containing supplements were at increased risk of having inadequate iodine intake [51], therefore also at increased risk of iodine deficiency. Furthermore, our findings that the pescatarians, flexitarians and omnivores, had a median UIC < 100 µg/l indicated inadequate intakes despite consumption of iodine-rich animal source foods, supports previous findings of mild iodine deficiency in young women in the general population of Norway [52]. Norway is still one of the countries that has voluntary salt iodization, which has been implemented in many countries to combat iodine deficiency globally [53]. Thus, the main sources of dietary iodine in Norway are lean fish, cow’s milk, dairy yoghurt, and eggs. Therefore, people who omit these food groups may be at particular risk of inadequate iodine intake. In the past decade, several population groups (women of fertile age, pregnant, breastfeeding women, infants who are exclusively breastfed, adult vegans, and immigrants) in Norway have been repeatedly found to have intakes below recommendations, showing that inadequate iodine intake is widespread [49, 52, 54]. For young women of reproductive age who exclude all animal dietary sources of iodine, it is particularly important that they obtain iodine from supplements or foods fortified with iodine [55]. If a pregnancy starts with low iodine stores, it can have consequences for the thyroid function in mothers, as well as neurodevelopment in their children [56].

### Multivariable adjustment

Multivariable adjustment of the energy-adjusted micronutrient intakes using gender and age as covariates was performed in our study, in which minor changes were observed. Thus gender and age did not seem to significantly influence the reported micronutrient intake in our study sample. Although the findings from the multivariable adjustment needs to be interpreted with caution as all the 95%CI were overlapping between the groups, except for total intake of iron. Increased uncertainties in the results are probably caused by fewer than 30 participants in the plant-based diet groups. Furthermore, we did observe that the participants with higher level of GNKQ had significantly higher intakes of vitamin A, folate, vitamin D, vitamin C and iron compared to those with poor GNKQ level. This finding also emphasizes the importance of securing food education to youth for them to have a well-planned diet that meets their dietary needs. Furthermore, this finding indicates that future studies assessing dietary intake among youth following plant-based diets compared to omnivores should account for other factors that may impact micronutrient adequacy, aside from their dietary pattern.

### Energy intake and misreporting

In our study, all dietary groups reported an energy intake below the lower energy requirements for females and males aged 18–24 years [39]. Thus, in this study, considerable underreporting is presumed, resulting in an underestimation of the micronutrient intake. Under-reporting is a well-known issue with self-reporting in dietary assessments [57]. In response to this, energy adjustments were performed for all micronutrients based on the nutrient density principle and according to individual energy requirements using the Henry equation to estimate the BMR and by using individual PAL values.

Intake estimates from 24-hour dietary recalls contain random within person error, particularly due to the day-to-day variation in the dietary intake. Thus, we need to acknowledge that there is a risk that participants might be wrongly categorized as having inadequate or adequate intakes when comparing individuals to AR. However, in this study, up to four non-consecutive 24-hour dietary recalls were evaluated, which can partially mitigate the concern about day-to-day variation. In addition, the 24-hour recalls were employed on both weekdays and weekends. We also assessed supplement use in both 24-hour dietary recalls and as habitual supplement use in the previous six months, strengthening the intake data on supplement use.

### Strengths and limitations

Our study has strengths and limitations. To our knowledge, this is the first study to evaluate micronutrient intake and status of youth who follows different plant-based diets compared to omnivores in Norway. Therefore, providing up-to-date data in this population group are warranted. A major strength was the use of blood and urine biomarkers as objective markers, in addition to self-reported dietary data. However, it needs to be noted that MMA was measured using DBS and the Hb in capillary blood, which might be less precise compared to when measured in venous blood. Though, when avoiding venipuncture, we also reduce the participation burden. A previous validation study have shown that MMA measured using DBS can be used as a robust measurement with high precision and accuracy [58]. Another strength was the use of a previously validated tool for dietary assessment (Myfood24) that uses image-assisted portion sizes for a more accurate estimate of portion sizes [59]. However, a limitation of the Myfood24 database was the limited availability of vegan/vegetarian food products, which could potentially be one of the explanations for the low reported energy intake. However, an open text field was available, which allowed reporting of food items that were not present in the database.

A limitation of the study is the small sample size in the different diet groups; as a result, it was not possible to perform statistical analysis split by sex, age group, or supplement users within the different diet groups. In addition, we were not able to perform statistical analysis evaluating intrapersonal variability, due to the fit of the data. However, when using repeated 24-hour recalls these data can be used to evaluate the proportion at risk of inadequate intake, although there is a risk of wrongly categorizing participants with higher proportion of inadequate or adequate intake [60]. Due to the sample size, there is a risk of type II error, and therefore our results need to be interpreted with this kept in mind. Another limitation is the recruitment method used, which was based on convenience sampling, thus a risk is recruitment of a non-representative sample, which would impact the generalizability of our study findings. We also need to acknowledge that the PAL values used in the energy adjustment of the nutrient intakes is based on self-reported physical activity data, which can inaccurately result in higher or lower individual energy requirements, which needs to be taken into consideration when interpreting the data. Another limitation was the use of six months as diet duration as inclusion criteria, as it might be insufficient diet duration for a deficiency of anemia and B_12_ deficiency to manifest. Although our study will provide an insight into the current situation in a youth population, which is lacking. Furthermore, this study took place during the COVID-19 pandemic, which might have influenced recruitment and possibly their dietary intake. However, a report by the Norwegian Institute of Public Health [61], found that Norwegian adolescents did not change their diet to a considerable extent prior to, during, or after the COVID-19 pandemic, except those already consuming a higher sugar intake than recommended, as they reported an additional increase in sugary foods and sugary beverages.

## Conclusion

Our findings show from the total intake including food and supplements (energy-adjusted) that both youth with plant-based and omnivorous diets faced nutritional challenges in meeting AR. Particularly for selenium, vitamin D and iodine. UIC supported increased risk of inadequate iodine intake, with mild iodine deficiency across all diet groups and moderate iodine deficiency in vegans. Due to the small sample size in our study, there is a risk of type II error, thus, our findings should be replicated in a larger sample examining dietary intake and status of youth, both those who follow plant-based and omnivore diets. Until then, we recommend that the iodine status of youth, especially young fertile women who omit all sources of dietary iodine, should be closely monitored until a mandatory salt iodization program is implemented in Norway. We also recommend youth who excludes most dietary iodine sources to obtain iodine from supplements or foods fortified with iodine as currently recommended from the Norwegian health authorities. Furthermore, we recommend that food education on how to secure sufficient nutrients from food in general and particularly adequate intake of vitamin D, selenium and iodine should be provided to the youth population in Norway.

## Electronic supplementary material

Below is the link to the electronic supplementary material.

Supplementary Material 1

Supplementary Material 2

## Declarations

### Standards of reporting

A STROBE checklist for reporting observational studies has been provided with this submission.
