# Supplementary material for: Micronutrient intake and status in young vegans, lacto-ovo-vegetarians, pescatarians, flexitarians, and omnivores
Source: Eur J Nutr. 2024 Jul 18;63(7):2725–41. doi: 10.1007/s00394-024-03453-4 (PMC11490461; doi:10.1007/s00394-024-03453-4)
Supplement: Supplementary file 2 — Supplementary Material 2 [file 394_2024_3453_MOESM2_ESM.docx]

**Supplemental Table 1** Absolute intake of vitamins in 16-to-24-year-olds following different plant-based and omnivore diets.

| Absolute intakes^‖‖‖^ | All  n = 165 | | | Vegans  n = 19 | | | Lacto-ovo vegetarians  n = 20 | | | Pescatarians  n = 30 | | | Flexitarians  n = 25 | | | Omnivores  n = 71 | | | P |
| --- | --- | --- | --- | --- | --- | --- | --- | --- | --- | --- | --- | --- | --- | --- | --- | --- | --- | --- | --- |
|  | 50^th^ | | 25^th^, 75^th^ | 50^th^ | | 25^th^, 75^th^ | 50^th^ | | 25^th^, 75^th^ | 50^th^ | | 25^th^, 75^th^ | 50^th^ | | 25^th^, 75^th^ | 50^th^ | | 25^th^, 75^th^ |  |
| Vit A, RE/d, food^‡^ | 406 | 263, 558 | | 281^*^ | 182, 451 | | 390^§^ | 239, 466 | | 465^‖‖^ | 341, 646 | | 359^§^ | 254, 520 | | 451^§^ | 285, 622 | | **0.021** |
| Vit A, RE/d, total^‡^ | 915 | 610, 1408 | | 850 | 403, 1514 | | 777 | 616, 1020 | | 1075 | 647, 1499 | | 1043 | 638, 1388 | | 870 | 636, 1377 | | 0.64 |
| Vit B_3_, NE/d, food‡ | 13 | 9, 20 | | 12 | 8, 20 | | 8^*^ | 7, 13 | | 12 | 9, 17 | | 12 | 9, 22 | | 15^§^ | 11, 23 | | **0.002** |
| Vit B_3_, NE/d, total^‡^ | 15 | 10, 27 | | 32^*^ | 11, 41 | | 10^‖‖^ | 7, 20 | | 13^‖‖^ | 10, 23 | | 21 | 10, 29 | | 16 | 11, 26 | | **0.002** |
| Vit B_2_, mg/d, food | 1 | 1, 2 | | 1 | 1, 2 | | 2 | 1, 2 | | 1 | 1, 2 | | 1 | 1, 2 | | 2 | 1, 2 | | 0.16 |
| Vit B_2_, mg/d, total^‡^ | 2 | 1, 2 | | 3 | 1, 3 | | 1 | 1, 2 | | 2 | 1, 2 | | 2 | 1, 3 | | 2 | 1, 2 | | 0.14 |
| Vit B_12_, µg/d, food^‡^ | 4 | 2, 5 | | 1^*^ | 0, 1 | | 2^*,§^ | 1, 3 | | 4^§^ | 2, 5 | | 4^§^ | 3, 6 | | 5^‖‖^ | 3, 7 | | **<0.001** |
| Vit B_12_, µg/d, total^‡^ | 5 | 3, 7 | | 7 | 2, 15 | | 3 | 2, 5 | | 5 | 2, 6 | | 5 | 3, 8 | | 5 | 3, 7 | | 0.11 |
| Folate, µg/d, food^‡^ | 232 | 177, 292 | | 307^*^ | 245, 501 | | 234^§^ | 166, 314 | | 236 | 186, 300 | | 230^§^ | 169, 261 | | 214^§^ | 160, 270 | | **<0.001** |
| Folate, µg/d, total^‡^ | 258 | 192, 405 | | 497^*^ | 246, 615 | | 292 | 187, 343 | | 274 | 190, 419 | | 316 | 230, 574 | | 227^§^ | 172, 302 | | **<0.001** |
| Vit D, µg/d, food^‡^ | 3 | 1, 4 | | 1^*^ | 1, 3 | | 2 | 1, 4 | | 4^§^ | 1, 6 | | 3 | 1, 6 | | 3 | 2, 5 | | **0.005** |
| Vit D, µg/d, total^‡^ | 7 | 2, 18 | | 15 | 2, 28 | | 4 | 1, 20 | | 7 | 4, 16 | | 7 | 2, 14 | | 7 | 2, 14 | | 0.42 |
| Vit E, α-TE, food^‡^ | 11 | 8, 14 | | 12 | 10, 17 | | 9^§^ | 8, 11 | | 13^*^ | 10, 18 | | 9 | 7, 14 | | 11 | 8, 14 | | **0.005** |
| Vit E, α-TE, total^‡¶^ | 13 | 9, 19 | | 19 | 11, 25 | | 10 | 8, 12 | | 16 | 10, 22 | | 14 | 9, 23 | | 13 | 8, 18 | | **0.034** |
| Vit C, mg/d, food^‡^ | 76 | 41, 107 | | 102^*^ | 71, 159 | | 76 | 33, 98 | | 65 | 39, 93 | | 80 | 50, 103 | | 69^§^ | 29, 99 | | **0.035** |
| Vit C, mg/d, total^‡^ | 84 | 47, 137 | | 152^*^ | 71, 239 | | 83 | 45, 108 | | 72 | 43, 221 | | 92 | 71, 144 | | 79^§^ | 31, 123 | | **0.032** |

Abbreviations:50^th^, 25^th^, 75^th^ = percentiles, foods = micronutrients solely from foods, total = including foods and supplements; ^‖‖‖^Absolute intakes = not energy-adjusted intake from 1-4 repeated 24-hour dietary recalls using average value; ^‡^Test for the difference using Kruskal Wallis one-way ANOVA with correction for multiple comparisons, values with different superscript indicate statistically significant differences from post hoc test (^§,‖‖, *^)^;¶^ For vitamin E no difference between groups indicated by adjusted p-value; Statistically significant values between the dietary groups < 0.05 are given in bold.

**Supplemental Table 2** Absolute intake of mineral and trace elements in 16-to-24-year-olds following different plant-based and omnivore diets.

| Absolute intakes^‖‖‖^ | All  n = 165 | | Vegans^†^  n = 19 | | Lacto-ovo vegetarians^†^  n = 20 | | Pescatarians^†^  n = 30 | | Flexitarians^†^  n = 25 | | | Omnivores^†^  n = 71 | | P |
| --- | --- | --- | --- | --- | --- | --- | --- | --- | --- | --- | --- | --- | --- | --- |
|  | 50^th^ | 25^th^, 75^th^ | 50^th^ | 25^th^, 75^th^ | 50^th^ | 25^th^, 75^th^ | 50^th^ | 25^th^, 75^th^ | 50^th^ | | 25^th^, 75^th^ | 50^th^ | 25^th^, 75^th^ |  |
| Magnesium, mg/d, food^‡^ | 290 | 227, 368 | 411^*^ | 265, 678 | 257^§^ | 198, 308 | 300 | 234, 396 | 281 | 232, 330 | | 282 | 221, 353 | **0.019** |
| Magnesium, mg/d, total^‡^ | 316 | 235, 401 | 411^*^ | 296, 699 | 275^§^ | 208, 377 | 309 | 234, 417 | 316 | 262, 371 | | 316 | 228, 295 | **0.029** |
| Calcium, mg/d, food^‡^ | 662 | 472, 930 | 498 | 453, 1102 | 557 | 442, 698 | 723 | 531, 926 | 602 | 425, 896 | | 717 | 479, 1037 | 0.30 |
| Calcium, mg/d, total^‡^ | 712 | 500, 961 | 696 | 471, 1118 | 607 | 521, 724 | 726 | 538, 926 | 670 | 425, 990 | | 774 | 529, 1043 | 0.26 |
| Iron, mg/d, food^‡^ | 9 | 7, 12 | 13^*^ | 9, 18 | 8^§^ | 6, 10 | 8 | 7, 10 | 9 | 7, 10 | | 9^§^ | 7, 11 | **0.012** |
| Iron, mg/d, total^‡^ | 10 | 7, 16 | 18^*^ | 10, 28 | 10 | 7, 13 | 9 | 8, 15 | 11 | 8, 20 | | 10^§^ | 7, 12 | **0.010** |
| Zinc, mg/d, food^‡^ | 8 | 6, 11 | 7 | 6, 12 | 6^*^ | 5, 8 | 8 | 6, 10 | 7 | 6, 9 | | 9^§^ | 7, 12 | **<0.001** |
| Zinc, mg/d, total^‡^ | 9 | 7, 13 | 16^*^ | 7, 22 | 7^§^ | 5, 9 | 8 | 7, 12 | 9 | 7, 12 | | 9^*^ | 7, 12 | **0.004** |
| Selenium, µg/d, food^‡^ | 34 | 24, 49 | 30 | 18, 53 | 25^*^ | 17, 26 | 36^§^ | 28, 52 | 42^§^ | 20, 57 | | 37^§^ | 27, 51 | **<0.001** |
| Selenium, µg/d, total^‡^ | 41 | 25, 60 | 60^*^ | 19, 90 | 25^§^ | 18, 35 | 43 | 29, 68 | 50^*^ | 28, 125 | | 38 | 27, 52 | **0.012** |
| Iodine, µg/d, food^‡^ | 71 | 48, 109 | 35^*^ | 27, 43 | 62 | 47, 83 | 90^§^ | 57, 146 | 79^§^ | 48, 132 | | 79^§^ | 57, 118 | **<0.001** |
| Iodine, µg/d, total^‡^^¶^ | 41 | 26, 69 | 43 | 31, 182 | 64 | 53, 101 | 98 | 65, 211 | 102 | 58, 199 | | 79 | 58, 130 | **0.044** |

Abbreviations:50^th^, 25^th^, 75^th^ = percentiles, foods = micronutrients solely from foods, total = including foods and supplements; ^‖‖‖^Absolute intakes = not energy-adjusted dietary intake from 1-4 repeated 24-hour dietary recalls using average value; ^‡^ Test for the difference using Kruskal Wallis one-way ANOVA with correction for multiple comparisons, values with different superscript indicate statistically significant differences from post hoc test (^§^,^*^); **^¶^** In post hoc test, no difference indicated between groups by adjusted p-value; Statistically significant values between the dietary practices <0.05 are given in bold.

| Absolute intake |  |  |  |  | All^†^  <AR | | Vegans^†^  <AR | | Lacto-ovo-vegetarians ^†^  <AR | | | | Pescatarians^†^  <AR | | | Flexitarians^†^  <AR | | | Omnivores^†^  <AR | | |  |
| --- | --- | --- | --- | --- | --- | --- | --- | --- | --- | --- | --- | --- | --- | --- | --- | --- | --- | --- | --- | --- | --- | --- |
|  | Females,  16-17 years | Males,  16-17 years | Females,  18-24 years | Males,  18-24 years | n | % | n | % | n | % | | n | | % | n | | % | n | | % | P | |
| Vitamin A, RE/d^‡^ | 500 | 600 | 540 | 630 | 72 | 44 | 8 | 42 | 13 | **65** | 9 | | | 30 | 11 | | 44 | 31 | | 44 | 0.20 | |
| Niacin, NE/MJ^‡¶^ | 1.3 | 1.3 | 1.3 | 1.3 | 59 | 36 | 5 | 26 | 12 | **60** | 12 | | | 40 | 10 | | 40 | 20 | | 28 | 0.09 | |
| Riboflavin, mg/d^‡^ | 1.3 | 1.3 | 1.3 | 1.3 | 58 | 35 | 5 | 26 | 9 | 45 | 11 | | | 37 | 9 | | 36 | 24 | | 34 | 0.81 | |
| Vitamin B_12,_ µg/d^‡^ | 3.1 | 3.2 | 3.2 | 3.2 | 49 | 30 | 7 | 37 | 10 | **50** | 8 | | | 27 | 5 | | 20 | 19 | | 27 | 0.22 | |
| Folate, µg/d^‡^ | 240 | 250 | 250 | 250 | 79 | 48 | 5 | 26 | 8 | 40 | 13 | | | 43 | 10 | | 40 | 43 | | **61** | 0.05 | |
| Vitamin D, µg/d^‡^ | 7.5 | 7.5 | 7.5 | 7.5 | 92 | 56 | 8 | 42 | 13 | 65 | 16 | | | 53 | 14 | | 56 | 41 | | 58 | 0.69 | |
| Vitamin E, α-TE/d^‡^ | 9 | 10 | 8 | 9 | 14 | 9 | 1 | 5 | 2 | 10 | 1 | | | 3 | 1 | | 4 | 9 | | 13 | 0.58 | |
| Vitamin C, mg/d^‡^ | 75 | 85 | 75 | 90 | 91 | 45 | 5 | 26 | 9 | 45 | 16 | | | **53** | 7 | | 28 | 37 | | **52** | 0.09 | |
| Magnesium, mg/d^‡^ | 200 | 240 | 240 | 280 | 47 | 29 | 3 | 16 | 7 | 35 | 8 | | | 27 | 5 | | 20 | 24 | | 34 | 0.46 | |
| Calcium, mg/d^‡^ | 980 | 980 | 870 | 870 | 110 | 67 | 12 | 63 | 19 | 95 | 21 | | | 70 | 17 | | 68 | 41 | | 58 | **0.023** | |
| Iron, mg/d^‡^ | 9 | 9 | 9 | 7 | 59 | 36 | 3 | 16 | 8 | 40 | 14 | | | 47 | 9 | | 36 | 25 | | 35 | 0.28 | |
| Zinc, mg/d^‡^ | 10.2 | 11.7 | 8.1 | 10.6 | 77 | 47 | 6 | 32 | 13 | 65 | 15 | | | 50 | 12 | | 48 | 31 | | 44 | 0.31 | |
| Selenium, µg/d^‡^ | 55 | 70 | 60 | 70 | 126 | 76 | 10 | 53 | 18 | 90 | 21 | | | 70 | 16 | | 64 | 61 | | 86 | **0.006** | |
| Iodine, µg/d^‡^ | 100 | 110 | 120 | 120 | 109 | 66 | 12 | 63 | 17 | 85 | 16 | | | 53 | 13 | | 52 | 51 | | 72 | 0.07 | |

**Supplemental Table 3** Proportion of individuals below average requirement (AR) of 16-to-24-year-olds following different dietary practices (n=165).

^†^Percentage presented within each diet group below average requirement including food and supplements from absolute intake (not energy-adjusted), in which each individual are compared against The Nordic Nutrition Recommendation 2023 according to the average requirement value specific for their age and gender; ^‡^Test for the difference (categorical variables) using cross tabulation with Pearson Chi-Square and Fisher-Exact (two-sided); Statistically significant values between the dietary practices < 0.05 are given in bold.

**Supplemental Table 4** Proportion of individuals below average requirement (AR) of 16-to-24-year-olds by general nutrition knowledge level (GNKQ) (n=165).

| Including foods and supplements |  | |  |  |  | Poor GNKQ^†^  <AR | | Moderate GNKQ ^†^  <AR | | High GNKQ ^†^  <AR | |  |  |  |  |
| --- | --- | --- | --- | --- | --- | --- | --- | --- | --- | --- | --- | --- | --- | --- | --- |
|  | Females,  16-17 years | | Males,  16-17 years | Females,  18-24 years | Males,  18-24 years | n | % | n | % | n | % | | | P |  |
| Energy adjusted vitamin A, RE/d^‡^ | 500 | 600 | | 540 | 630 | 6 | 27 | 21 | 23 | 1 | 2 | | **<0.001** | | |
| Energy adjusted niacin, NE/MJ^‡¶^ | 1.3 | 1.3 | | 1.3 | 1.3 | 4 | 18 | 9 | 10 | 5 | 10 | | 0.49 | | |
| Energy adjusted riboflavin, mg/d^‡^ | 1.3 | 1.3 | | 1.3 | 1.3 | 2 | 9 | 11 | 12 | 6 | 12 | | 1.0 | | |
| Energy adjusted vitamin B_12,_ µg/d^‡^ | 3.1 | 3.2 | | 3.2 | 3.2 | 4 | 18 | 9 | 10 | 8 | 16 | | 0.36 | | |
| Energy adjusted folate, µg/d^‡^ | 240 | 250 | | 250 | 250 | 2 | 9 | 14 | 15 | 2 | 4 | | 0.12 | | |
| Energy adjusted vitamin D, µg/d^‡^ | 7.5 | 7.5 | | 7.5 | 7.5 | 16 | **73** | 41 | 45 | 18 | 35 | | **0.012** | | |
| Energy adjusted vitamin E, α-TE/d^‡^ | 9 | 10 | | 8 | 9 | 0 | 0 | 1 | 1 | 0 | 0 | | 1.0 | | |
| Energy adjusted vitamin C, mg/d^‡^ | 75 | 85 | | 75 | 90 | 8 | 36 | 27 | 29 | 6 | 12 | | **0.021** | | |
| Energy adjusted magnesium, mg/d^‡^ | 200 | 240 | | 240 | 280 | 2 | 9 | 3 | 3 | 0 | 0 | | 0.11 | | |
| Energy adjusted calcium, mg/d^‡^ | 980 | 980 | | 870 | 870 | 9 | 41 | 30 | 33 | 16 | 31 | | 0.69 | | |
| Energy adjusted iron, mg/d^‡^ | 9 | 9 | | 9 | 7 | 2 | 9 | 5 | 5 | 1 | 2 | | 0.28 | | |
| Energy adjusted zinc, mg/d^‡^ | 10.2 | 11.7 | | 8.1 | 10.6 | 7 | 32 | 7 | 8 | 3 | 6 | | **0.006** | | |
| Energy adjusted selenium, µg/d^‡^ | 55 | 70 | | 60 | 70 | 14 | **64** | 52 | **57** | 28 | **55** | | 0.82 | | |
| Energy adjusted iodine, µg/d^‡^ | 100 | 110 | | 120 | 120 | 10 | 46 | 40 | 44 | 20 | 39 | | 0.88 | | |

^†^Percentage presented within each level of GNKQ below average requirement, in which each individuals are compared against The Nordic Nutrition Recommendation 2023 according to the average requirement value specific for their age and gender; ^§^ Poor GNKQ = total GNKQ sum score <60% correct, moderate GNKQ = total GNKQ sum score correct answers between 60-79%, high GNKQ = sum score 80-100% correct answers (previously described in details elsewhere, Groufh-Jacobsen et al., 2023); ^‡^Test for the difference (categorical variables) using cross tabulation with Pearson Chi-Square and Fisher-Exact (two-sided); Statistically significant values between level of GNKQ < 0.05 are given in bold.

**Supplemental Table 5** English translation of the items used in the electronic questionnaire in VeggiSkills-Norway

| Item category | Questions | Answer options |
| --- | --- | --- |
| Part 1: Sedentary behavior, physical activity, tobacco use, and sleep behavior | | |
| Sedentary behavior | 1. How many hours do you typically spend sitting during a 24-hour period?   Reflect on the past six months (including at work/school, transportation, TV, reading, PC use, etc.) | 1. Choose the most suitable number of hours by using the scale and enter the value (ranging from 0-24 hours). |
| Physical activity | 1. How often do you engage in physical activity?   For example, brisk walking, skiing, cycling, swimming, or other sports/activities where you get out of breath and sweat. Take an average over the past six months.   1. For how long are you usually physically active?   By physical activity, we mean when you get out of breath and sweat.  Take an average over the past six months. | 1. Never / Less than once a month/ once a week/ 2-3 times a week/ 4-5 times a week/ almost every day 2. Less than 15 minutes/ 15-29 minutes/ 30-60 minutes/more than 60 minutes |
| Snuff and smoking | 1. How often do you use snuff and/or smoke? Reflect on the past six months. 2. If you specified "other" for tobacco use, please specify. | \| Frequency \| Never \| rarely \| occasionally \| Daily \| \| --- \| --- \| --- \| --- \| --- \| \| Snuff \|  \|  \|  \|  \| \| Cigarettes \|  \|  \|  \|  \| \| E-cigarettes \|  \|  \|  \|  \| \| Other tobacco \|  \|  \|  \|  \|  1. Tick of one option per row. 2. Open text option |
| Sleeping behavior | 1. How many hours do you usually sleep per day on weekdays? Reflect on the past six months. 2. How many hours do you usually sleep per day on weekends? Reflect on the past six months. | 1. Choose the number of hours using the scale and enter the value (ranging from 0-24 hours). 2. Choose the number of hours using the scale and enter the value (ranging from 0-24 hours). |
| Part 2. Dietary habits | | |
| Dietary habits | 1. How often do you include the following food items in your diet? Reflect on the past six months. 2. Do you avoid certain foods or drinks because of… 3. If you selected "other" for reasons to avoid certain foods/drinks, please specify: | 1. Tick of one option per row  \| Food item \| Never \| Rarely \| Occasionally \| Often \| \| --- \| --- \| --- \| --- \| --- \| \| Milk or dairy products (cow/goat) \|  \|  \|  \|  \| \| Eggs (including in food/cooking) \|  \|  \|  \|  \| \| Fish or fish products \|  \|  \|  \|  \| \| Poultry (e.g., turkey, chicken, duck) \|  \|  \|  \|  \| \| Meat or meat products \|  \|  \|  \|  \|  1. Tick of one option per row (Tick of "No" if you do not avoid the item).  \| Reason \| No \| Occasionally \| Yes \| \| --- \| --- \| --- \| --- \| \| Allergy or intolerance \|  \|  \|  \| \| My health \|  \|  \|  \| \| My religion \|  \|  \|  \| \| Dislike the taste \|  \|  \|  \| \| My friends \|  \|  \|  \| \| My family \|  \|  \|  \| \| My weight \|  \|  \|  \| \| Better appearance \|  \|  \|  \| \| Climate concerns \|  \|  \|  \| \| Animal welfare \|  \|  \|  \| \| Economy \|  \|  \|  \| \| Other \|  \|  \|  \|  1. Open text option |
| Self-reported dietary practice | 1. Indicate your diet. Reflect on the past six months. If you recently changed your diet, select the one you followed for the past six months. 2. If you selected "other" diet, please specify: | 1. Omnivore/ Flexitarian/ Pescatarian/ Lacto-ovo-vegetarian/ Lacto-vegetarian/ Ovo-vegetarian/ Vegan/ Not sure/ Other diet      1. Open text option |
| Meal pattern | 1. How often do you eat the following meals during a typical week? Reflect on the past six months.   For more information see detailed description in previous paper (Groufh-Jacobsen S, et al., 2023) | 1. Tick of one option per row.  \| Meal \| Rarely/Never \| 1 time per week \| 2 times per week \| 3 times per week \| 4 times per week \| 5 times per week \| 6 times per week \| \| --- \| --- \| --- \| --- \| --- \| --- \| --- \| --- \| \| Breakfast \|  \|  \|  \|  \|  \|  \|  \| \| Lunch \|  \|  \|  \|  \|  \|  \|  \| \| Dinner \|  \|  \|  \|  \|  \|  \|  \| \| Evening meal \|  \|  \|  \|  \|  \|  \|  \|  \|  \| Rarely/Never \| 1  time per week \| 2-4 times per week \| 5-6 times per week \| 1 time per day \| 2-3 times per day \| 4-5 times per day \| 6+ times per day \| \| --- \| --- \| --- \| --- \| --- \| --- \| --- \| --- \| --- \| \| Snacks \|  \|  \|  \|  \|  \|  \|  \|  \| |
| Dietary screener | 1. How often do you consume the following food items?   Reflect on the past six months.  For more information see detailed description in previous paper (Groufh-Jacobsen S, et al., 2023) | 1. Tick of one option row.  \| Food Item \| Rarely/  Never \| 1 time per month \| 2-3 times per month \| 1 time per week \| 2-4 times per week \| 5-6 times per week \| 1 time per day \| 2-3 times per day \| \| --- \| --- \| --- \| --- \| --- \| --- \| --- \| --- \| --- \| \| Sweetened/partially sweetened breakfast cereal (e.g., Special K, Cornflakes) \|  \|  \|  \|  \|  \|  \|  \|  \| \| Unsweetened breakfast cereal (e.g., oatmeal, 4-grain, Weetabix) \|  \|  \|  \|  \|  \|  \|  \|  \| \| Whole grain bread/crispbread/rolls (>50% whole grain) \|  \|  \|  \|  \|  \|  \|  \|  \| \| White cheese, all types \|  \|  \|  \|  \|  \|  \|  \|  \| \| Brown cheese/prim \|  \|  \|  \|  \|  \|  \|  \|  \| \| Yogurt/buttermilk based on cow’s milk, all types \|  \|  \|  \|  \|  \|  \|  \|  \| \| Whole grain pasta, barley rice, whole grain couscous, etc. \|  \|  \|  \|  \|  \|  \|  \|  \| \| Cow’s milk, all types \|  \|  \|  \|  \|  \|  \|  \|  \| \| Plant-based milk alternatives, all types \|  \|  \|  \|  \|  \|  \|  \|  \| \| Vegetables, including salad, cabbage, carrots, green beans, etc. (excluding potatoes and sweet potatoes) \|  \|  \|  \|  \|  \|  \|  \|  \| \| Beans, lentils, chickpeas, peas (excluding green beans) \|  \|  \|  \|  \|  \|  \|  \|  \| \| Fruits and berries of all types, including fresh, frozen, and canned (excluding juice or smoothie) \|  \|  \|  \|  \|  \|  \|  \|  \| \| Juice/smoothie (excluding nectar) \|  \|  \|  \|  \|  \|  \|  \|  \| \| Potatoes/sweet potatoes (baked, boiled, mashed) \|  \|  \|  \|  \|  \|  \|  \|  \| \| Fried potatoes/sweet potatoes (French fries) \|  \|  \|  \|  \|  \|  \|  \|  \| \| Unsalted nuts (walnuts, hazelnuts, almonds, etc.) and seeds \|  \|  \|  \|  \|  \|  \|  \|  \| \| Salted snacks (popcorn, chips, salted nuts) \|  \|  \|  \|  \|  \|  \|  \|  \| \| Fish spread (e.g., mackerel in tomato, herring) \|  \|  \|  \|  \|  \|  \|  \|  \| \| Fatty fish (salmon, mackerel, etc.) \|  \|  \|  \|  \|  \|  \|  \|  \| \| Lean fish (cod, saithe, etc.) \|  \|  \|  \|  \|  \|  \|  \|  \| \| Fish products (fish cake, fish sticks, etc.) \|  \|  \|  \|  \|  \|  \|  \|  \| \| Red meat, ground or pieces (beef, lamb/sheep, pork, goat) \|  \|  \|  \|  \|  \|  \|  \|  \| \| Processed meat (hamburger, sausage, kebab, etc.) \|  \|  \|  \|  \|  \|  \|  \|  \| \| Plant-based ready-made products (all types of meat substitutes) \|  \|  \|  \|  \|  \|  \|  \|  \| \| Pizza, all types \|  \|  \|  \|  \|  \|  \|  \|  \| \| Tomato sauce, including salsa for taco, ketchup, pasta sauce, etc. (excluding pizza) \|  \|  \|  \|  \|  \|  \|  \|  \| \| Sweets, including chocolate (including vegan) \|  \|  \|  \|  \|  \|  \|  \|  \| \| Cakes, buns, biscuits, waffles, ice cream, etc. (including vegan) \|  \|  \|  \|  \|  \|  \|  \|  \| \| Soft drinks, syrup, and nectar with sugar \|  \|  \|  \|  \|  \|  \|  \|  \| \| Sugary energy drinks (e.g., Gatorade, Red Bull) \|  \|  \|  \|  \|  \|  \|  \|  \| \| Coffee/tea/iced tea (only specify if with sugar/syrup/honey) \|  \|  \|  \|  \|  \|  \|  \|  \| \| Alcoholic beverages \|  \|  \|  \|  \|  \|  \|  \|  \| |
| Supplement use | 1. a) Have you used cod liver oil or cod liver oil capsules in the past six months?   b) This section is displayed only if you selected "Less than weekly," "1-2 times a week," "3-4 times a week," "5-6 times a week," or "Daily" in the previous question. Please specify the brand/type and amount you use of cod liver oil or cod liver oil capsules (dose/drops/number of capsules)   1. a) Have you used multivitamin supplements in the past six months?   b) This section is displayed only if you selected "Less than weekly," "1-2 times a week," "3-4 times a week," "5-6 times a week," or "Daily" in the previous question. Please specify the brand/type and amount you use of multivitamin.   1. a) Have you used vitamin D supplements (excluding cod liver oil and multivitamin supplements) in the past six months?   b) This section is displayed only if you selected "Less than weekly," "1-2 times a week," "3-4 times a week," "5-6 times a week," or "Daily" in the previous question. Please specify the brand/type and amount you use of vitamin D supplements.   1. a) Have you used beta-carotene supplements (excluding multivitamin supplements) in the past six months?   b) This section is displayed only if you selected "Less than weekly," "1-2 times a week," "3-4 times a week," "5-6 times a week," or "Daily" in the previous question. Please specify the brand/type and amount you use of beta-carotene supplement.   1. a) Have you used folate supplements (excluding multivitamin supplements) in the past six months?   b) This section is displayed only if you selected "Less than weekly," "1-2 times a week," "3-4 times a week," "5-6 times a week," or "Daily" in the previous question. Please specify the brand/type and amount you use of folate supplements.   1. a) have you used vitamin B12 supplements (excluding B12 injections or multivitamin supplements) in the past six months?   b) This section is displayed only if you selected "Less than weekly," "1-2 times a week," "3-4 times a week," "5-6 times a week," or "Daily" in the previous question. Please specify the brand/type and amount you use of B12 supplements.   1. a) Do you use B12 injections?   b) Please specify the time since your last injection (in years).  This section is displayed only if you selected "Yes" in the previous question.   1. a) Have you used nutritional yeast supplements in the past six months?   b) This section is displayed only if you selected "Less than weekly," "1-2 times a week," "3-4 times a week," "5-6 times a week," or "Daily" in the previous question. Please specify the brand/type and amount you use of nutritional yeast.   1. a) Have you used iodine supplements (excluding multivitamin supplements or seaweed supplements) in the past six months?   b) This section is displayed only if you selected "Less than weekly," "1-2 times a week," "3-4 times a week," "5-6 times a week," or "Daily" in the previous question. Please specify the brand/type and amount you use of iodine supplements.  c) Did you use iodine supplements yesterday or today? This section is displayed only if you selected "1-3 times a month," "1-2 times a week," "3-4 times a week," "5-6 times a week," or "Daily" in the previous question.   1. a) Have you used seaweed supplements in the past six months?   b) This section is displayed only if you selected "Less than weekly," "1-2 times a week," "3-4 times a week," "5-6 times a week," or "Daily" in the previous question. Please specify the brand/type and amount you use of seaweed.  c) Did you use seaweed supplements yesterday or today? This section is displayed only if you selected "1-3 times a month," "1-2 times a week," "3-4 times a week," "5-6 times a week," or "Daily" in the previous question.   1. a) Have you used iron supplements (excluding multivitamin supplements) in the past six months?   b) This section is displayed only if you selected "Less than weekly," "1-2 times a week," "3-4 times a week," "5-6 times a week," or "Daily" in the previous question. Please specify the brand/type and amount you use of iron | 1. a) No/ Less than weekly/ 1-2 times a week/ 3-4 times a week/ 5-6 times a week/ Daily 2. Open text option 3. a) No/ Less than weekly/ 1-2 times a week/ 3-4 times a week/ 5-6 times a week/ Daily   b) Open text option   1. a) No/ Less than weekly/ 1-2 times a week/ 3-4 times a week/ 5-6 times a week/ Daily   b) Open text option   1. a) No/ Less than weekly/ 1-2 times a week/ 3-4 times a week/ 5-6 times a week/ Daily   b) Open text option   1. a) No/ Less than weekly/ 1-2 times a week/ 3-4 times a week/ 5-6 times a week/ Daily   b) Open text option   1. a) No/ Less than weekly/ 1-2 times a week/ 3-4 times a week/ 5-6 times a week/ Daily   b) Open text option   1. a) no/yes   b) answer options ranging from 3 months to previous 3 years   1. a) No/ Less than weekly/ 1-2 times a week/ 3-4 times a   week/ 5-6 times a week/ Daily  b) Open text option  9 a) No/ Less than weekly/ 1-2 times a week/ 3-4 times a  week/ 5-6 times a week/ Daily  b) Open text option  c) No/yes   1. a) No/ Less than weekly/ 1-2 times a week/ 3-4 times a week/ 5-6 times a week/ Daily   b) Open text option  c) No/yes  11 a) No/ Less than weekly/ 1-2 times a week/ 3-4 times a  week/ 5-6 times a week/ Daily  b) Open text option |
| Part 3 food literacy competencies | | |
| Food skills | 1. Do you shop for your groceries yourself? 2. Who usually does the grocery shopping for you? (This question appears only if “no” or “sometimes” is selected in the previous question) 3. How often do you eat take-away or ready-made meals? 4. How often do you cook at home for yourself or others? | 1. no/sometimes/ yes, always 2. Open text option      1. Never/ Less than weekly/ Once a week/ 2-3 times a week/ 4-5 times a week/ Almost every day 2. Never/ Less than weekly/ Once a week/ 2-3 times a week/ 4-5 times a week/ Almost every day |
| General nutrition knowlegde (GNKQ) | For English original items see Kilemann, et al., 2016). In addition, see supplemental material in Groufh-Jacobsen et al., 2023 for adaption of the revised GNKQ questionnaire by Kilemann et al. to the population and into a Norwegian context, and also for information on the translation and back translation of the items. |  |
| Critical Nutrtion literacy (CNL) | CNL items used are developed by Guttersrud, O et al., 2014. See method section in Groufh-Jacobsen et al., 2023 for detailed description.  I have confidence in the various diets that I read about in newspapers, magazines, etc. (original item 24)  I am critical of the dietary information that I receive from various sources in society (original item 21)  I am concerned that the dietary information that I read may not be based on science (original item 20)  I am confident that the media’s presentation of new scientific findings concerning a healthy diet is correct (original item 29)  I am familiar with the criteria for scientifically based content in health claims (original item 23)  I often refer to newspapers and magazines if I discuss diet with others (original item 22)  I am influenced by the dietary advice that I read about in newspapers, magazines, etc. (original item 26)  I am confident that some of the methods within alternative medicine (such as health foods) provide me with credible dietary advice (original item 27)  I find it hard to distinguish scientific nutritional information from non-scientific nutritional information (original item 28)  I base my diet on information that I get from scientifically recognized literature (for instance, the journals published by the Norwegian Medical Association and the Norwegian Directorate of Health) (original item 30) | Answer options for all items:  disagree strongly/disagree partly/neither agree nor disagree/agree partly/agree strongly/not sure |
| Sources used for seeking nutrition information | 1. a) If you are looking for information about food/nutrition, where do you get this information from? Multiple choices possible.   b) If the alternative option ‘other’ was reported, an open-ended response was possible | a) Dietitian or doctor or health nurses / personal trainer or dietary advisor (did not include dietitian) / family or friends / influencers / Snapchat / Instagram / documentaries / mass media / books / food companies / the Norwegian Health Authorities / other  b) Open text option |
| Part 4 background | | |
| Background | 1. What year were you born? 2. What month were you born? 3. a) Gender   b) If you selected the "other" option under gender   1. How tall are you? 2. How much do you weigh now? 3. a) In which country were you born?   b) If you selected the "other" option under country   1. a) What is your native language?   b) If you selected the "other" option under native language   1. a) Where do you reside?   b) If you selected the "other" option under where are you reside   1. a) Which area in Kristiansand do you reside in?   b) If you selected the "other" option   1. a) Parental guardian (mother) – educational level   b) If you selected the "other" option   1. a) Parental guardian (father) – educational level   b) If you selected the "other" option   1. a) What best describes your current life situation?   b) If you selected the "other" option   1. a) What is your current employment situation? Multiple choices are possible. You must choose at least one option.   b) If you selected the "other" option   1. a) What is your current living situation?   b) If you selected the "other" option   1. How many people in your household are younger than 18 years old?   If you are under 18, include yourself. | 1. Option ranging from 1994-2007 2. Options ranging from January to December. 3. a) Female/ male/ other   b) Open text option   1. Answer in cm. 2. Answer in kg. 3. a) Norway / other   b) Open text option   1. a) Norwegian / other   b) Open text option   1. a) Kristiansand / other   b) Open text option   1. a) Kvadraturen / Eg / Grim / Lund / Randesund / Hånes / Tveit / Ålefjær / Justvik / Mosby / Stray / Gimlekollen / Vågsbygd / Tinnheia / Hellemyr / Slettheia / Flekkerøy / Søgne / Sogndal / Other, Kristiansand   b) Open text option   1. a) Less than 13 years of schooling (did not complete high school) /13 years of schooling (completed high school) / College/university, less than 4 years / College/university, 4 years or more / Other or not applicable.   b) Open text option   1. a) Less than 13 years of eucation (did not complete high school) / 13 years of education(completed high school) / College/university, less than 4 years / College/university, 4 years or more / Other or not applicable   b) Open text option   1. a) Single / In a relationship / Engaged or married / Other   b) Open text option   1. a) Student / Working full-time / Working part-time / Unemployed/Job-seeking / Other   b) Open text option   1. a) Living alone / Living in shared housing with several others / Living with a partner (friend/significant other/spouse) / Living with my guardian(s) / Other   b) Open text option   1. Open text option |

^†^ The questionnaire is employed in Norwegian language, and for English translation of this questionnaire an AI tool was used ChatGPT3, OpenAI. For full description of the dietary screener (“MyFoodMonth 1.1” based on Salvesen et al., 2023), general nutrition knowledge questionnaire (GNKQ) (based on Kilemann et al., 2014, GNKQ-R) and the critical nutrition literacy items (developed by Guttersrud et al., 2014) see previously published paper Groufh-Jacobsen et al., 2023.
